# Investigating Nanoscale Interactions of Host–Guest Complexes Formed Between CB[7] and Atenolol by Quantum Chemistry and Ultrasensitive Vibrational Spectroscopy

**DOI:** 10.3390/s24227156

**Published:** 2024-11-07

**Authors:** Anca Onaciu, Valentin Toma, Rareș-Mario Borșa, Vasile Chiș, Gabriela-Fabiola Știufiuc, Carina Culic, Constantin-Mihai Lucaciu, Rareș-Ionuț Știufiuc

**Affiliations:** 1 Department of NanoBioPhysics, Institute of Medical Research and Life Sciences—MEDFUTURE, “Iuliu Haţieganu” University of Medicine and Pharmacy, Louis Pasteur 4-6, 400349 Cluj-Napoca, Romania; valentin.toma@umfcluj.ro (V.T.); rares.mari.borsa@elearn.umfcluj.ro (R.-M.B.); 2 Department of Pharmaceutical Physics & Biophysics, Faculty of Pharmacy, “Iuliu Hatieganu” University of Medicine and Pharmacy, Louis Pasteur 6, 400349 Cluj-Napoca, Romania; clucaciu@umfcluj.ro; 3 Dental Medicine Faculty, “Iuliu Hatieganu” University of Medicine and Pharmacy, Pasteur 4, 400349 Cluj-Napoca, Romania; 4 Department of Maxillofacial Surgery and Implantology, “Iuliu Hațieganu” University of Medicine and Pharmacy, Cardinal Iuliu Hossu 37, 400029 Cluj-Napoca, Romania; 5 Department of Prosthetic Dentistry and Dental Materials, Division Dental Propaedeutics, Aesthetics, Dental Medicine Faculty, “Iuliu Hatieganu” University of Medicine and Pharmacy, Clinicilor 32, 400001 Cluj-Napoca, Romania; 6 Faculty of Physics, Babeş-Bolyai University, M. Kogălniceanu 1, 400084 Cluj-Napoca, Romania; vasile.chis@ubbcluj.ro (V.C.); gabriela.stiufiuc@ubbcluj.ro (G.-F.Ș.); 7 Department of Conservative Odontology, Division Odontology, Endodontics, Cariology, Oral Pathology, “Iuliu Hatieganu” University of Medicine and Pharmacy, Moților 33, 400089 Cluj-Napoca, Romania; culic@elearn.umfcluj.ro; 8 Nanotechnology Laboratory, TRANSCEND Research Center, Regional Institute of Oncology, 2-4 General Henri Mathias Berthelot Street, 700483 Iaşi, Romania

**Keywords:** SERS, beta-blockers, enantiomers, solid plasmonic substrate, CB[7]

## Abstract

In addition to the course of over 20 years of cucurbit-7-uril (CB[7]) in the pharmaceutical industry, the present study brings together the most recent observations from the perspective of ultrasensitive Raman spectroscopy and Density Functional Theory (DFT) related to the interaction of this molecule with atenolol (Ate) enantiomers during the formation of these host–guest complexes. Quantum chemistry calculations based on DFT were first used to understand the interaction geometry between CB[7] and Ate. These results were further confirmed by ultrasensitive vibrational spectroscopy. The spectral features associated with each enantiomer in the presence of CB[7] were analyzed by means of SERS, highlighting distinct interaction profiles. These experimental findings validated quantum chemical calculations, offering a comprehensive understanding of the host–guest interactions at the nanoscale level.

## 1. Introduction

Cucurbiturils (CB[n]s, n—number of glycoluril subunits) are macrocycles that can form strong inclusion complexes with other molecules through molecular recognition processes. Their centrally positioned hydrophobic cavity offers accommodation for the cationic guest molecule through the electronegative portal, which presents symmetry on the top and bottom due to hydrophilic carbonylated rims [1]. This geometry ensures a barrier for the guest molecule association and dissociation based on its size. Such thermodynamically stable complexes can be exploited in various applications where they can act as delivery platforms for controlled drug release [2,3], media neutralizers [4], and stereoselective and chiral separators [5].

In drug delivery applications, cucurbiturils present superior properties compared to cyclodextrins, since they can achieve better quantitative binding of the guest molecules due to their high affinity, especially in the case of protonated drug molecules, which leads to the formation of very stable complexes [6,7,8]. However, depending on the number of glycoluril subunits, some CBs are too small (CB[5]) while others are too large (CB[10]) to encapsulate drug molecules [1]. For these reasons, the most widely used members of the cucurbituril family are CB[6], CB[7], and CB[8]. The complexation of molecules with these CBs creates more soluble and resistant structures than noncomplexed molecules [9]. In addition, this host–guest binding system can enhance the bioactivity and bioavailability of the guest molecules by improving drug formulation and preventing its degradation [1]. Since the non-covalent interactions formed are characterized by weakness and reversibility, guest molecules can easily be released, usually during dilution in the new media [10]. However, other methods can be applied involving environmental pH modification or competitive binding of other molecules that are present in the media [10,11,12].

**Figure 1:**
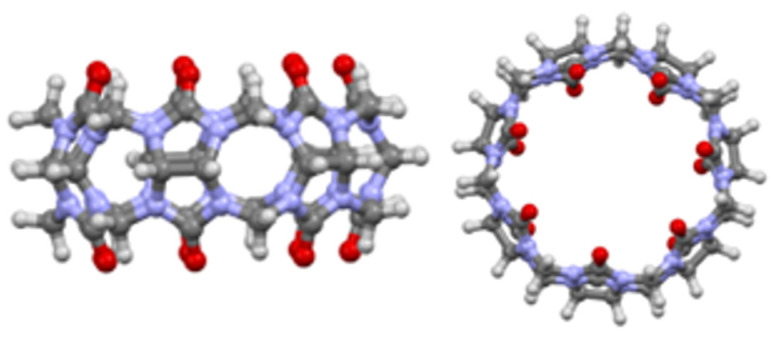
CB[7] molecule chemical structure (side view—left, top view—right); color code: red—oxygen, blue—nitrogen, gray—carbon, light gray—hydrogen).

CB[7] molecule (Figure 1) was first reported in the early 2000s. It is composed of seven glycoluril subunits, being part of the cucurbituril compounds family [13]. With an odd number of glycoluril subunits, CB[7] has a better water solubility than other family members that have an even number of subunits and need encapsulation or the addition of various salts [14]. It can encapsulate different molecules, especially molecules carrying cationic groups and even hydrophobic molecules based on its high affinity for such guests [15]. This process depends on the size and shape of the guest molecule and its chemical interactions with the electronegative portals, which can happen via van der Waals forces, or dipole–dipole or dipole–ion bonds [16,17]. In this regard, one of the first applications referred to drug delivery strategies using CB[7] as a vehicle for various molecules such as: oxaliplatin and biotin [18], albendazole [19], alkaloids [20], fluoroquinolone drugs [21], steroidal blocking agents [22], or vitamins [23].

The addition of functional groups like antibodies, peptides, or fluorescent molecules has enhanced the targeting features of the complexes and improved the imaging techniques [24,25,26,27,28,29]. It was revealed that CB[7] can boost the fluorescence intensity of fluorescent probes by improving their photostability [30,31]. In this regard, numerous other studies have reported the use of CB[7] in sensing applications [32,33,34,35]. Tandem enzyme assays were developed in order to monitor the activity of different molecules using spectroscopic methods [36,37]. CB[7]-dye conjugates were applied for drug detection in various fluids [33], cellular imaging [29], protein interaction studies [38], or heavy metal recognition [39].

Having the capacity to recognize different molecules, CB[7] was exploited in the field of chiral separation methods too [40,41,42,43]. Chiral separation of pharmaceutical active substances is of critical importance both for the pharmaceutical industry and for the medical field. It is usually performed through chromatography and electrophoresis methods, most likely via high-liquid performance chromatography [44,45,46]. Many studies have exploited the properties of cucurbiturils’ cavity in chirality sensing [47,48]. Some of them reported successful results in enantiomeric discrimination between R and S drug enantiomers [49], respectively diastereomeric separation between various L and D dipeptides [50]. The molecule encapsulation phenomena is usually analyzed through UV-Vis spectroscopy, proton nuclear magnetic resonance spectroscopy (1H NMR), density functional theory (DFT), and computational methods [51,52].

**Figure 2:**
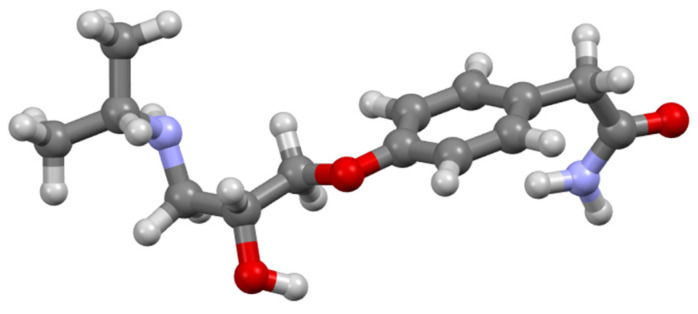
Atenolol molecule chemical structure; color code: red—oxygen, gray—carbon, light gray—hydrogen, blue—nitrogen.

It has been demonstrated through NMR spectroscopy that some beta-blockers like Atenolol (Ate) (Figure 2) can form 1:1 host–guest complexes with CB[7]. The CB[7] molecule has an outer diameter of around 16 Å, while the inner cavity diameter is approximatively 7.3 Å [53]. Due to these dimensions, Ate can bind over the central phenyl ring, which can be fully incorporated into the hydrophobic cavity of CB[7] [54].

On the other hand, according to a literature search, the theoretical calculations for quantifying the migration of enantiomers of Ate into the cavity of macrocycles was performed only in the case of cyclodextrins, indicating a superior selectivity for S-Atenolol [55]. In addition, our group has previously investigated the interactions between certain beta-blocker enantiomers and cyclodextrins by combining DFT computations with Surface Enhanced Raman Spectroscopy (SERS) [56,57,58,59,60,61].

In the pharmaceutical industry, analytical methods based on Raman spectroscopy started to be implemented for the quantitative and qualitative analysis of drug molecules due to their simplicity, reproducibility, time efficiency, and costs, which make them superior to chromatography methods [62,63,64]. Raman is engaged in the analysis of crystalline or solid forms of various drugs having the capacity to determine the pharmaceutical formulation purity [65].

Raman and SERS analyses can be employed to monitor single-molecule complexation with CB[7] [66,67]. The CB[7] and CB[8] macromolecules have the capacity to form reproducible hot-spot regions when used as a spacer between adjacent substrate nanoparticles [68,69,70,71]. Several drug isomers were investigated based on host–guest size matching using self-assembled plasmonic substrates made of gold nanoparticles conjugated with CB[7] [66,72].

In this study, our aim is to investigate the interactions between the CB[7] macrocycle with R and S Ate enantiomers by means of SERS. The geometry of interaction responsible for the formation of the host–guest complexes between CB[7] and the two enantiomers of Ate was first assessed by quantum chemistry calculations. SERS analysis of the complexes performed on solid plasmonic substrates possessing a high degree of spectral reproducibility confirmed the theoretical findings. Also, through this research our purpose is to fill the major gaps existing in the fascinating field of supramolecular interactions in order to achieve a better understanding of the interplay between molecular structure and interaction dynamics. The capacity of CB[7] to discriminate between R-(+) and S-(−) Ate, proved by SERS spectroscopy, was dependent on complexes’ concentration and could imply different adsorption geometries of the complexes on the solid plasmonic substrates used for recording the experimental spectra.

## 2. Materials and Methods

### 2.1. DFT Computations

Molecular geometry optimizations and Raman spectra calculations were performed using the Gaussian 16, revision C.01 software package. We employed density functional theory (DFT) with the wB97XD exchange functional. The basis set 6-311+G(d,p) was used for geometry optimizations of individual molecules, while for ATE-CB7 complexes (for geometry optimizations, Raman spectra and interaction energy calculations) we used the ONIOM scheme [73] at the wB97XD/6-311+G(d,p):wB97XD/3-21G level of theory. A two-layer ONIOM QM:QM’ scheme was used, where the real system included all the atoms and is calculated at the wB97XD/3–21G level of theory (QM’), while the model system, calculated at the wB97XD/6–311+G(d,p) level of theory (QM), contained only the Ate molecule.

### 2.2. Synthesis and Characterization of Colloidal Silver Nanoparticles 

Silver nanoparticles were synthesized according to the Leopold and Lendl method [74]. The method involved the reduction of silver nitrate using hydroxylamine. Briefly, 17 mg AgNO_3_ (International Laboratory, Cluj-Napoca, Romania) were dissolved in 10 mL ultrapure water (18.2 MΩ·cm, ELGA Labwater from PURELAB Chorus, Buckinghamshire, UK) and this solution was added over a 90 mL aqueous solution composed of 10.5 mg HONH_2_·HCl (VWR Chemicals, Leuven, Belgium) and 12 mg NaOH (International Laboratory, Cluj-Napoca, Romania) via magnetic stirring conditions. The obtained colloids were filtered using a tangential flow filtration procedure (Minimate™ TFF system from Pall Corporation, New York, NY, USA) in order to remove the byproducts and to concentrate them [75,76,77].

Physico-chemical characterization involved UV-Vis absorption spectroscopy and transmission electron microscopy (TEM). The UV-Vis absorption spectrum was recorded in the 300–800 nm wavelength interval and a step size of 1 nm using Duetta™ Fluorescence and Absorbance Spectrometer (HORIBA Scientific, Kyoto, Japan). TEM was employed to analyze the shape and the size of the silver nanoparticles. These measurements were performed on a Hitachi HT7700 microscope (Hitachi, Tokyo, Japan) equipped with an 8-megapixel XR81 camera (AMT, Woburn, MA, USA) operating at 80 kV. The diluted sample drop was placed on top of a carbon film-coated copper grid of mesh 200 (Ted Pella Inc., Redding, CA, USA) and incubated for approximately 20 min. Then the excess of liquid was blotted away using filter paper and the grid was placed in a desiccator until TEM analysis. The resulting images were processed using ImageJ software (National Institutes of Health, New York, NY, USA) [78].

### 2.3. Samples Preparation for SERS Analysis

CB[7] (Strem Chemicals Incorporated, Newburyport, MA, USA), R-(+) and S-(−) atenolol ((±)-4-[2-Hydroxy -3-[(1-methylethyl)amino]propoxy]benzeneacetamide) (Sigma Aldrich, St Louis, MO, USA) working solutions of 1 mM and 0.1 mM were prepared by dissolving the specific amounts in ultrapure water. The supramolecular systems were prepared at a 1:1 stoichiometry ratio by mixing equivalent volumes of CB[7] solution with each enantiomer solution. The mixtures were incubated for 24 h at 4°C to form the complexes.

The previously prepared colloids were then used as the main building blocks for the preparation of the solid plasmonic substrates using an original procedure developed in our laboratory [77]. The silver nanoparticles were deposited on the CaF_2_ Raman grade glass (Crystran Ltd., Poole, UK), which was heated to 40 °C to obtain solid plasmonic substrates. On top of these solid substrates, the working samples were deposited and allowed to dry at room temperature prior to spectra recording. All the measurements were conducted on the same batch of silver colloids.

SERS measurements were performed on a confocal Renishaw^®^inVia microscope (Renishaw plc, Wotton-under-Edge, Gloucestershire, UK), equipped with a Leica microscope (Leica Microsystems GmbH, Wetzlar, Germany), having a spectral resolution <1 cm^−1^. An internal silicon reference was used to calibrate the wavelength. All spectra were collected using 50× objective. A laser of 785 nm excitation with 1.95 mW power at the sample surface was used. The acquisition time was set to 5 s, and for each sample were recorded two maps of 50 points in different positions across it. Baseline correction was applied to eliminate the fluorescence background using Wire 4.2 software from Renishaw. The spectra were processed with the help of the OriginPro ^®^ 2019 software.

## 3. Results

### 3.1. DFT Calculations

**Figure 3:**
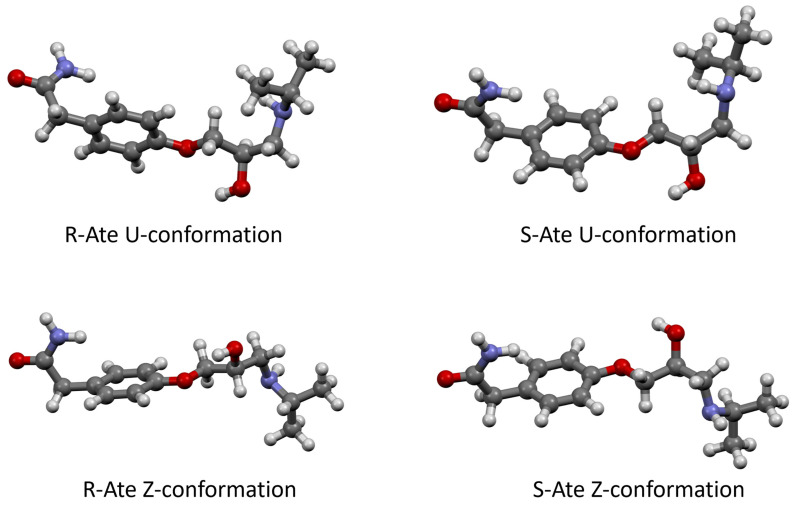
Optimized geometries of R/S Ate from DFT calculations. Atom color representation code: Black—Carbon, White—Hydrogen, Red—Oxygen and Blue—Nitrogen, according to Robert Corey, Linus Pauling, and Walter Koltun [79,80].

According to quantum chemistry calculations, Ate enantiomers present two geometrically distinct types of conformations when they are alone and not complexed with other molecules, namely Z-type and U-type. The chemical structure and the optimized geometries of Z and U conformations were calculated using the wB97XD method at 6-311+G(d,p) level of theory and are presented in Figure 3. Of these, the most stable one was determined to be Z-type for both enantiomers (Table 1).

**Figure 4:**
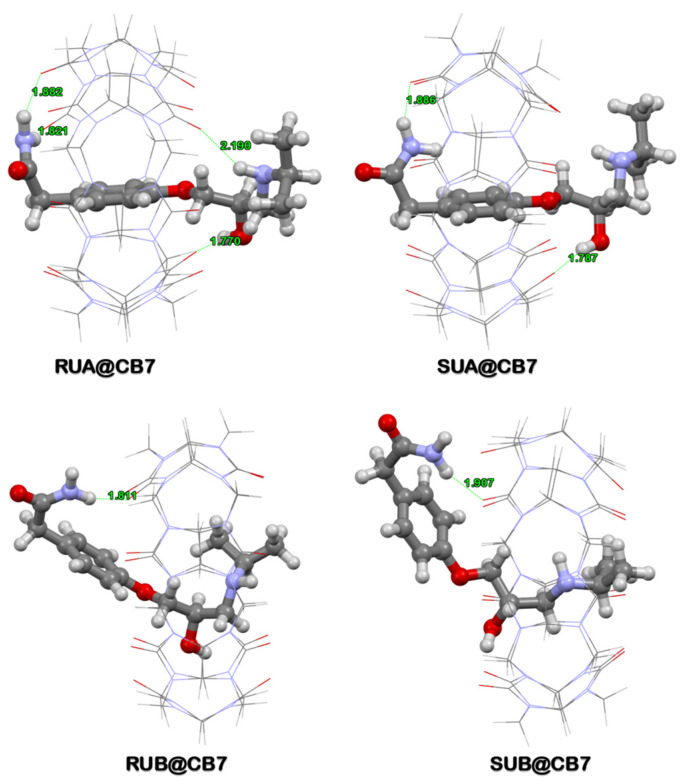
A- and B-type complexes formed between the most stable U-type R/S-Ate and CB[7] according to DFT calculations at the ONIOM (wB97XD/6–311+G(d,p):wB97XD/3–21G) level of theory.

When associated with CB[7], U-type Ate conformation generated complexes with the highest degree of stability as compared to Z-type. This happened because the U-type conformers can establish H-bonds with the CB[7] rims at both ends. Moreover, the complexes formed can present two distinct conformations (A and B) as shown in Figure 4.

One can observe that in the case of A-type conformation, the phenyl ring of Ate is positioned in the middle of the CB[7] cavity, while in the case of B-type conformation, the Ate phenyl ring stands outside the CB[7] cavity. In addition, the A forms of the U-type complexes are the ones that present the best integration of Ate into the CB[7] cavity with similar complexation energies (−35.03 kcal/mole for R-Ate, −36.62 kcal/mole for S-Ate). Moreover, as observed in Figure 4, four H-bonds are formed for the RUA@CB[7] complex, two of them being realized through the amino group and two through the OH and NH groups. Instead, for the SUA@CB[7] complex, only two H bonds are formed, one through the amino group and one through the hydroxyl group. This fact can explain the increased stabilities of the R-Ate enantiomers.

The Cartesian coordinates for all these structures can be found in Table S1 in Supplementary Information.

### 3.2. Synthesis and Characterization of SERS Substrates 

Silver colloid synthesis involved the chemical reduction of silver nitrate using hydroxylamine to obtain silver nanoparticles. These were then used to obtain solid plasmonic substrates and to analyze the interactions between CB[7] macrocycle and atenolol enantiomers. All the results coming from physico-chemical characterization can be found in Supplementary Information (SI) files. Physico-chemical characterization of silver nanoparticles involved the analysis of optical properties and morphological features. Silver colloids presented a maximum absorbance at 402 nm (Figure S1). Nanoparticles’ sizes were found to be between 30–50 nm, according to TEM images (Figure S2).

### 3.3. SERS Measurements

**Figure 5:**
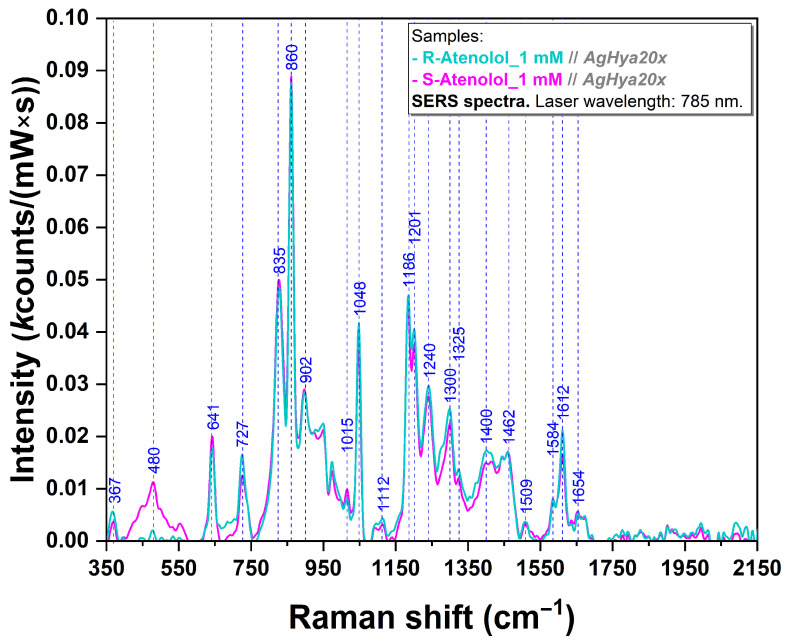
SERS spectrum of R-Ate (turquoise) and S-Ate (magenta) at 1 mM concentration using an excitation wavelength of 785 nm.

The SERS spectra of pure R/S-Ate were recorded using the same experimental conditions. As expected, the spectra are almost identical (Figure 5), being dominated by the following vibrational bands: 641, 727, 835, 860, 1048, 1186, 1201, and 1612 cm^−1^.

**Figure 6:**
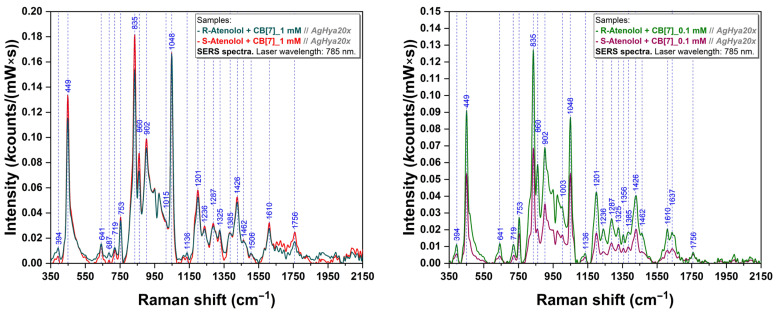
SERS spectra of R-Ate@CB[7] and S-Ate@CB[7] complexes at 1 mM (**left**) and 0.1 mM (**right**) concentrations.

Then, the formation of the complexes between CB[7] macrocycles and Ate enantiomers was investigated by SERS. In the case of complexes formed between CB[7] and R-Ate (R-Ate@CB[7]), respectively S-Ate (S-Ate@CB[7]) we have recorded spectra at two concentrations: 1 mM and 0.1 mM respectively. For both concentrations and types of complexes the bands located at 449, 835, and 1048 cm^−1^ stand out as being the most intense ones (Figure 6). For a solution concentration of 1 mM, minor intensity differences can be noticed in the case of S-Ate@CB[7] as compared to R-Ate@CB[7] (Figure 6 (left)). On the other hand, the results are very different for 0.1 mM concentrations (Figure 6 (right)), where noticeable amplification can be observed regarding the spectrum of R-Ate@CB[7] complex with respect to S-Ate@CB[7]. These results suggest that the CB[7] molecule could be explored for chiral sensing potential via SERS using host–guest complexes. In a previous study we used quite a similar approach to discriminate Propranolol enantiomers by means of SERS using cyclodextrins as host molecules [60].

**Figure 7:**
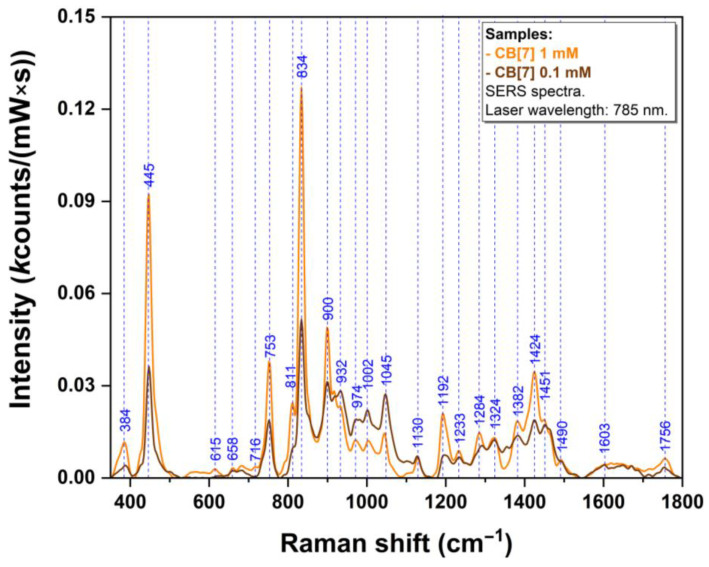
SERS spectrum of CB[7] 1 mM (orange) and 0.1 mM (brown) solutions.

The SERS spectra of pure CB[7] molecules at two solution concentrations (1 and 0.1 mM respectively) are presented in Figure 7. Very surprisingly, since we analyzed a molecule with quite a good affinity for the metallic substrate, the spectra recorded for analyte concentrations of 0.1 and 1 mM, respectively, are quite different with respect to vibrational band intensities and ratios. The spectra are dominated by several bands (445, 753, 834, 900, 1192, and 1424 cm^−1^).

For a better understanding of the spectral signature of the studied molecules, Table 2 and Table 3 provide a tentative assignment of the vibrational bands presented in the recorded spectra together with the calculated Raman wave numbers from DFT analysis.

## 4. Discussion

The present study represents a fundamental investigation on the macrocycle–drug complexes in terms of evaluating the interactions between the host (CB[7]) and the guest (R/S-Ate) from the perspective of DFT and SERS. DFT modeling was engaged to evaluate the potential of the CB[7] molecule to form complexes with Ate enantiomers, while ultrasensitive vibrational spectroscopy offered new insights regarding the nanoscale interactions between these complexes and silver solid plasmonic substrate.

For the SERS analysis, silver nanoparticle-based substrates developed by our group were used. These substrates proved their reproducibility in terms of experimental analysis of various classes of molecules [77,85,86,87]. The method of sample deposition on a solid substrate was preferred to the incubation method for a few reasons. The incubation method presents several drawbacks to be considered in terms of random and unstable spatial orientation of the analyte molecules on the SERS substrate. This leads to disruptive intermolecular reactivity between analyte and substrate nanoparticles on the acquired spectral results. In other words, the deposition of the analyte on the solid plasmonic substrate ensures the reproducibility of the experimental conditions and the related results, creating the premises for the development of innovative methods of analysis of pharmaceutical formulas.

All SERS measurements were done in the same setup and quantum chemistry calculations were used for an accurate band assignment for R/S Ate, CB[7] (Table 2) and their complexes (Table 3).

For quantum chemistry calculations, the DFT WB97XD method with a 6-311+G(d,p) level of theory was used because it has been shown to be the best possible at describing H-bond based interactions [88]. Atenolol molecule geometries proved various distinct conformations (Figure 3) from which Z-type conformation turned out to be the most stable (Table 1).

The SERS spectra acquired on the enantiomers of atenolol revealed almost identical spectra, which entitles us to emphasize their almost identical behavior of interaction with the silver plasmonic substrate (Figure 5). Various shifts can be observed when comparing experimental vibrational bands’ values with those obtained from DFT calculations. These differences are due to distinct interaction of Ate enantiomers with the silver plasmonic substrate, as was shown by Farcas et al. [56]. Most of the vibrational bands correspond to phenyl ring vibrational modes (Table 2). The bands located at 641 (in plane ring deformation, δ(CCC ring), τ(N_5_H_2_) [56,65,83]), 727 (ω(CCC ring), ρ(N_5_H_2_), δ(NH) [56,65]), 835 (γ(CH ring), ρ(N_5_H_2_), δ(CCC ring) [65,66,68]), 860 (ν(C_7_C_9_) [56,65,83]), 1048 (*ρ*(N_5_H_2_) [56]), 1186 (ν(C_13_C_18_), ω(C18H2), ν(CCC) [56,65]), 1201 (ν(C6O7), δ(CH ring), ν(CC ring), δ(CH2), δ(CCC) [65,67]), 1612 cm^−1^ (ν(CC ring), δ(CH ring), δ (NH_2_) [56,65,83]) were the most evident.

Complex simulations revealed that U-type conformers offer the best stability for the interactions between CB[7] and Ate enantiomers. As can be seen in Figure 4, the two Ate enantiomers form a 1:1 host–guest complex with the CB[7] molecule, with the phenyl ring being located inside of the CB[7] cavity in the case of A-type complexation. However, these interactions are not identical; in the case of S-Ate, the number of H-bonds are different with respect to R-Ate. This very subtle difference can be experimentally proven only in the case of 0.1 mM concentration (Figure 6 (right)). Furthermore, this aspect significantly recommends exploiting SERS analysis for chiral discrimination perspectives in the case of low concentration analytes.

For a higher concentration (1 mM) the complexes will be forced to have a similar interaction with the plasmonic substrate and therefore no significant intensity-related differences can be detected in their SERS signatures (Figure 6 (left)). On the other hand, it is important to mention that the Raman signal of 1 mM complexes is more amplified than in the case of 0.1 mM complexes.

On a first visualization of the SERS spectra recorded on the complexes, the vibrational bands located at 449, 835, and 1048 cm^−1^ are noted. According to quantum chemistry calculations these bands were located as 433 (δ(N-C-N) CB[7], δ(H3C-CH-CH3), ρ(acetamide) R-Ate, γ(ring), δ(H3C-CH-CH3) S-Ate)), 830 (δ(NCN) CB[7])), and 1040/1041 cm^−1^ (horizontal scissoring CB[7]).

Concerning CB[7] spectra, the bands located at 445 and 834 cm^−1^ (assigned to a ring scissoring mode and ring breathing mode respectively [66,81]) are the most “meaningful” ones. When a molecule is inserted inside the CB[7] cavity, these modes undergo modifications and their shift could represent strong evidence of this encapsulation, as shown in literature [66]. By comparing SERS spectra of the complexes (Figure 6) with those of CB[7] (Figure 7), a shift from 445 cm^−1^ to 449 cm^−1^ can be observed, suggesting the interactions of R/S-Ate with both portals of CB[7]. The 834 cm^−1^ vibration band (ρ(CH_2_) [66,81,82]) from the equatorial region of CB[7] and the 835 cm^−1^ band in the case of complexes rely on the interaction of CB[7] with the plasmonic substrate. It is well known that CB[7] has a good affinity for metallic surfaces through the portal carbonyl groups [89]. According to quantum chemistry calculations, this band was assessed at 832 and 830 cm^−1^, respectively, and corresponds to (N-C-N) group bending vibration. The differences observed in the case of SERS spectra of pure CB[7] (0.1 mM & 1 mM, respectively; Figure 7) suggest a distinct spatial orientation of these molecules when attached to the solid plasmonic substrate. When the concentration is higher (1 mM), the CB[7] molecules will be forced to have a similar interaction with the substrate. This type of interaction is also valid for the complexes formed between CB[7] and Ate enantiomers. This is the reason why the differences between the complexes formed with the two enantiomers of Ate are more visible for a 0.1 mM concentration.

All together, these results show different patterns of interactions between host and guest molecules from a theoretical and an experimental approach, which support each other in providing quality information.

## 5. Conclusions

In this study we have employed quantum chemistry calculations combined with SERS analysis of the host–guest complexes in order to investigate the nanoscale interactions between CB[7] macrocycle and R/S-Ate enantiomers. DFT analysis contributed in helping us understand the binding interactions arising between CB[7] molecules and the two enantiomers of Atenolol, and reflected the formation of stable complexes with similar energies of interaction. Among these, RUA@CB[7] conformers resulted in having an increased stability due to the four H-bonds established between R-Ate and CB[7] as compared to S-enantiomer which form only two H-bonds with the host molecule. SERS spectroscopy was able to experimentally prove this very slightly higher affinity of CB[7] macrocycle for the R enantiomer of atenolol. It turned out that host–guest complexes’ concentration plays a major role in nanoscale detection of these differences, with the best results being observed for 0.1 mM.

We truly believe that this type of combined quantum chemistry/SERS analysis holds the key for unraveling nanoscale molecular interactions that could also be used for other applications such as chiral discrimination.

## Figures and Tables

**Table 1:** Calculated relative free energies and Boltzmann populations for R/S-Ate conformers.

Conformer	ΔG (kcal/mol)	Boltzmann Factor	Boltzmann Populations (%)
R-Ate U	0.851	0.238	9.60%
**R-Ate Z**	0.000	1.000	**40.40%**
S-Ate U	0.851	0.238	9.60%
**S-Ate Z**	0.000	1.000	**40.40%**

**Table 2:** Experimental and calculated vibrational band wave numbers of CB[7] and R/S-Ate together with a tentative assignment.

Experimental SERS Vibrational Band (cm^−1^)	Calculated Raman Vibrational Band (cm^−1^)	Molecular Group Vibration of R/S-Ate and CB[7] Individual Spectra (Literature)	Molecular Group Vibration of R/S-Ate and CB[7] Individual Spectra (DFT Computations)
367	368	*ρ*(N_5_H_2_), *ρ*(C_18_H_2_) [56]	γ(ring-O), δ(CCNH_2_)
445	434	σ (ring) [66,67,81,82]	Vertical δ(N-C-N)
480	-	*ρ*(C_9_H_2_) [56]	-
641	653	in plane ring deformation, δ(CCC ring), τ(N_5_H_2_) [56,65,83]	δ(CCC ring), τ(NH_2_)
658	660	τ(HC–CH) [82]	Vertical scissoring
727	742	ω(CCC ring), ρ(N_5_H_2_), δ(NH) [56,65]	γ(ring)
753	753	-	γ(C=O) in CB[7]
834 /835	832 /841	γ(CH ring), ρ(N_5_H_2_), δ(CCC ring) in R/S-Ate [56,83,84] Ring breathing, δ(C–N–C), ρ(CH_2_) in CB[7] [66,81,82]	γ(ring) in R/S-Ate δ(N-C-N) in CB[7]
860	863	ν(C_7_C_9_) in R/S-Ate [56,65,83]	ρ(CH_3_), β(NH) in R/S-Ate
900 /902	898 /886	Ring deformation in R/S-Ate [84] β(C–N–C), τ(N–C–C–N), ν(C–C) in CB[7] [82]	Ring breathing in R/S-Ate ρ(CH_2_) in CB[7]
974	950/968	-	ν(CC)ring, ρ(CH_2_) in CB[7]
1045 /1048	1052 /1054	*ρ*(N_5_H_2_) in R/S-Ate [56]	ν(O-CH_2_), ρ(CH_3_) in R/S-Ate horizontal scissoring in CB[7]
1186	1179	ν(C_13_C_18_), ω(C_18_H_2_), ν(CCC) [56,65]	ν(C_ring_-O), δ(CCC ring), ω(CH_2_)
1192	1207	-	ν(H_2_C-N)
1201	1196	ν(C_6_O_7_), δ(CH ring), ν(CC ring), δ(CH_2_), δ(CCC) [65,83]	τ(CH_2_), δ(OH)
1233	1247	-	ν(C-N) in CB[7]
1240	1250	δ(O_10_H_2_), δ(C_11_H_2_), δ(N_12_H_2_), *ν*(O_1_C_12_), *δ*(CCC ring) [56,83]	ω(CH_2_)
1300	1305	*δ*(C_19_N_5_H_40_), *ω*(C_18_H_2_) [56]	δ(OCNH_2_), δ(CCC ring)
1324 /1325	1341/1326	δ(CH_2_),ν(CH), δ(CH_3_), ν(CH_3_), δ(C_8_H_23_), ω(C_11_H_3_) in R/S-Ate [56,83]	ν(CN), τ(CH_2_) in CB[7]
1382	1402	-	γ(CH), ω(CH_2_), ν(CN)
1400	-	ν(CN), νas(CN)[84]	-
1424	1425/1444	δ(CH_3_), δ(C_11_H_2_), δ(C_8_H_2_), δ(C_18_H_2_), δ(N_5_H_2_), v(CCC) in R/S-Ate [56,65,83] νas(CN) in CB[7] [82]	δ(COH), ω(CH_2_) in R/S-Ate β(CH_2_) in CB[7]
1462	1464	-	δ(CH_3_)
1584	1572	-	β(NH_2_)
1612	1615	ν(CC ring), δ(CH ring), δ(NH_2_) [56,65,83]	ν(CC ring)
1756	1757	-	In plane ν(C=O)

ν: stretching mode; ν as: antisymmetric stretching; δ: bending mode; γ: out of plane bending; ρ-rocking; ω: wagging; τ: twisting; σ: scissoring. Color wave numbers text legend: blue–vibrational bands for CB[7], red–vibrational bands for R/S-Ate, black–common vibrational bands.

**Table 3:** Experimental and calculated vibrational band wave numbers of R/S-Ate@CB[7] complexes together with a tentative assignment from DFT computations.

Experimental SERS Vibrational Band (cm^−1^)	Calculated Raman Vibrational Band (cm^−1^)	Molecular Group Vibration of R/S-Ate@CB[7] Complexes (DFT Computations)
394	395	ρ(ring), deformation(methythyl-amino-propoxy) in R/S-Ate
449	433	δ(N-C-N) in CB[7] δ(H_3_C-CH-CH_3_), ρ(N_5_H_2_) in R-Ate γ(ring), δ(H_3_C-CH-CH_3_) in S-Ate
641	644	vertical scissoring in CB[7] σ (ring), β(OH) in R/S-Ate
719	704/706	δ(NCN),ρ(CH_2_) in R/S-Ate
753	745/744	γ(C=O) in CB[7] in plane ring deformation in R-Ate γ(ring) in S-Ate
835	830	δ(NCN) in CB[7]
860	856/858	Ring breathing, δ(H_2_C-CO-NH_2_) in R/S-Ate
902	888	ρ(CH_2_) in CB[7]
1048	1040/1041	Horizontal scissoring in CB[7]
1201	1213/1205	ν(C_ring_-O) in R-Ate ν(Cring-CH_2_) in S-Ate
1236	1233	ν(C-N) in CB[7]
1287	1295/1296	in plane ring deformation, τ(CH_2_), ω(CH_2_) in R/S-Ate
1325	1327	ν(C-N), τ(CH_2_) in CB[7]
1385	1397	γ(CH), w(CH_2_), ν(CN) in CB[7]
1426	1425/1424	ν(CC ring) in R/S-Ate β(CH_2_) in CB[7]
1610	1596	ν(CC ring) in R/S-Ate
1637	1630/1632	ν(CC ring) in R/S-Ate

## Data Availability

Data is contained within the article or Supplementary Material.

## Supplementary Materials

The following supporting information can be downloaded at: https://www.mdpi.com/article/10.3390/s24227156/s1, Figure S1: UV-Vis absorption spectrum of silver nanoparticles (AgHya) 20× concentrated using TFF method; Figure S2: Size distribution and TEM image of the 20× concentrated silver nanoparticles; Table S1: Cartesian coordinates of CB[7], R/S-Ate, R/S-Ate@CB[7].
