# Supplementary material for: Investigating Nanoscale Interactions of Host–Guest Complexes Formed Between CB[7] and Atenolol by Quantum Chemistry and Ultrasensitive Vibrational Spectroscopy
Source: Sensors (Basel). 2024 Nov 7;24(22):7156. doi: 10.3390/s24227156 (PMC11598021; doi:10.3390/s24227156)
Supplement: Supplementary file 1 [file sensors-24-07156-s001.zip › sensors-3256937-supplementary.pdf]

Supplementary Information

Investigating nanoscale interactions of host–guest complexes formed between CB[7] and atenolol by quantum chemistry and ultrasensitive vibrational spectroscopy

Anca Onaciu [1,2*], Valentin Toma [1], Rareș-Mario Borșa [1,2,3,4,5], Vasile Chiș [6], Gabriela-Fabiola Ştiufiuc [6], Carina Culic [7], Constantin-Mihai Lucaciu[2], Rareș-Ionuț Ştiufiuc [1,2,8*]

1 Department of NanoBioPhysics, Institute of Medical Research and Life Sciences - MEDFUTURE, "Iuliu Hațieganu" University of Medicine and Pharmacy, Louis Pasteur 4-6, 400349 Cluj-Napoca, Romania; anca.onaciu@umfcluj.ro; valentin.toma@umfcluj.ro

2 Department of Pharmaceutical Physics & Biophysics, Faculty of Pharmacy, "Iuliu Hatieganu" University of Medicine and Pharmacy, Louis Pasteur 6, 400349 Cluj-Napoca, Romania; rares.stiufiuc@umfcluj.ro

3 Dental Medicine Faculty, "Iuliu Hatieganu" University of Medicine and Pharmacy, Pasteur 4, 400349 Cluj-Napoca, Romania; rares.mari.borsa@elearn.umfcluj.ro

4 Department of Maxillofacial Surgery and Implantology, "Iuliu Hațieganu" University of Medicine and Pharmacy, Cardinal Iuliu Hossu 37, 400029 Cluj-Napoca, Romania

5 Department of Prosthetic Dentistry and Dental Materials, Division Dental Propaedeutics, Aesthetics, Dental Medicine Faculty, "Iuliu Hatieganu" University of Medicine and Pharmacy, Clinicilor 32, 400001 Cluj-Napoca, Romania

6 Faculty of Physics, Babeş-Bolyai University, M. Kogălniceanu 1, 400084 Cluj-Napoca, Romania; vasile.chis@ubbcluj.ro ; gabriela.stiufiuc@ubbcluj.ro

7 Department of Conservative Odontology, Division Odontology, Endodontics, Cariology, Oral Pathology, "Iuliu Hatieganu" University of Medicine and Pharmacy, Moților 33, 400089 Cluj-Napoca, Romania; culic@elearn.umfcluj.ro

8 Nanotechnology Laboratory, TRANSCEND Research Center, Regional Institute of Oncology, 2-4 General Henri Mathias Berthelot Street, 700483 Iaşi, Romania.

* Correspondence: anca.onaciu@umfcluj.ro (A.O.) ; rares.stiufiuc@umfcluj.ro (R.-I.S.)

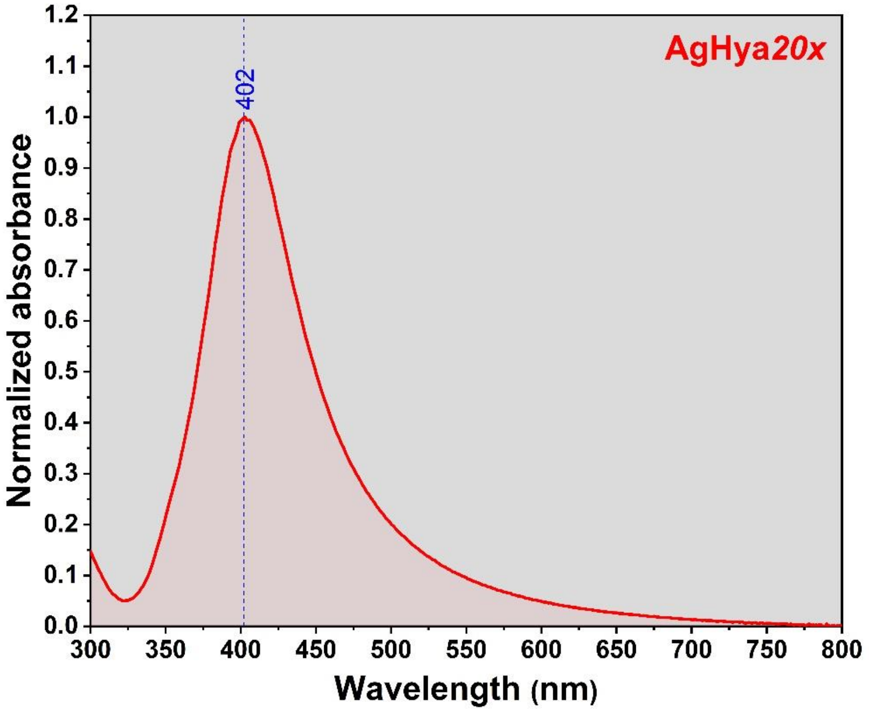

Figure S1. UV-Vis absorption spectrum of silver colloids after 20× concentration using the TFF method

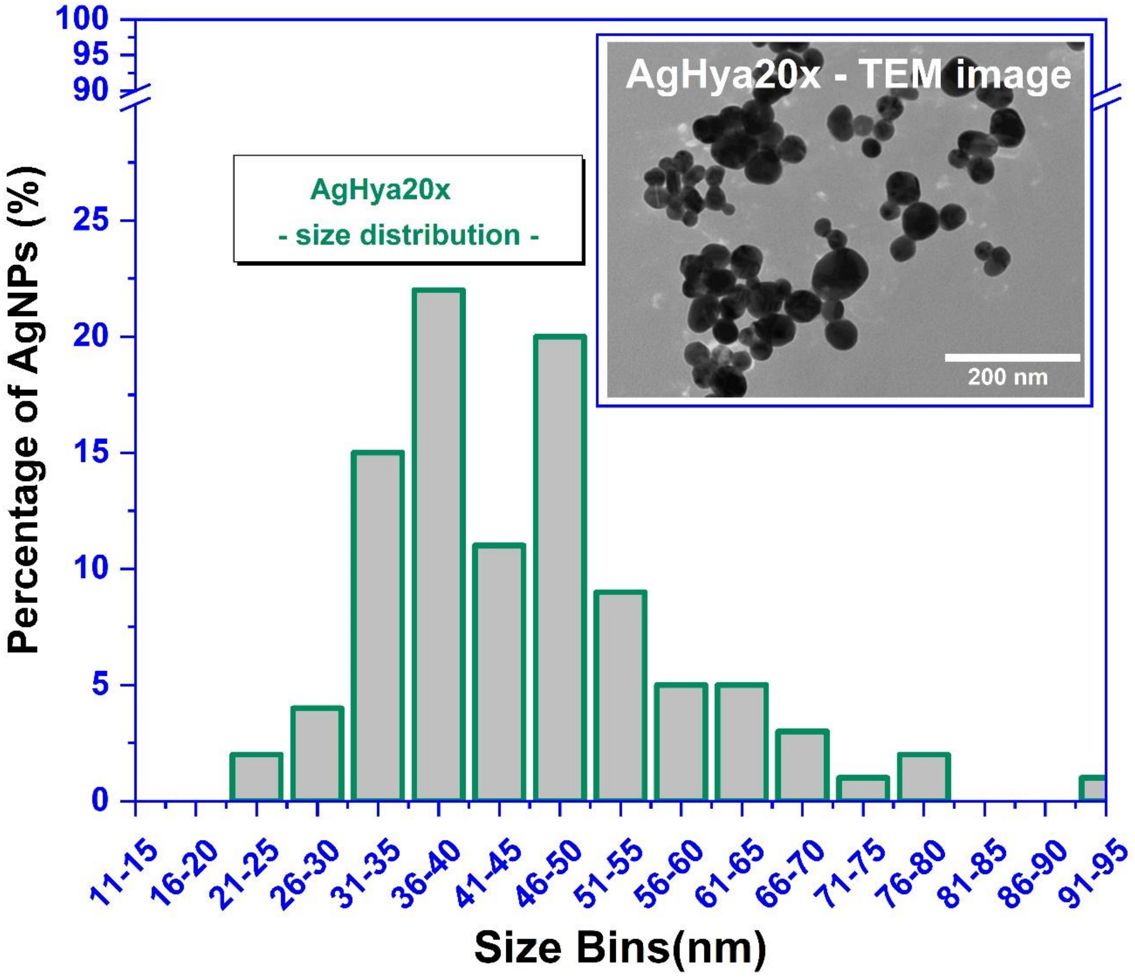

Figure S2. Size distribution and TEM image of the concentrated silver nanoparticles

Table S1. Cartesian coordinates of CB[7], R/S-Ate, R/S-Ate@CB[7]

Density functional: ωB97XD

CB7 D7h symmetry, gas-phase, 6-311+G(d,p) basis set

C	0.00000000	4.78165093	1.97563111
C	0.00000000	4.78165093	-1.97563111
C	0.00000000	-5.75046822	1.75224003
C	0.00000000	-5.75046822	-1.75224003
C	0.77799290	5.78602093	-0.00000000
C	1.80951301	-5.55058320	0.00000000
C	2.07468058	-4.30811863	1.97563111
C	2.07468058	-4.30811863	-1.97563111

C	2.49503465	5.18099284	1.75224003
C	2.49503465	5.18099284	-1.75224003
C	3.21140778	-4.87546626	0.00000000
C	3.73844524	2.98131059	1.97563111
C	3.73844524	2.98131059	-1.97563111
C	4.03862268	4.21578439	0.00000000
C	4.49589709	-3.58535829	1.75224003
C	4.49589709	-3.58535829	-1.75224003
C	4.66176496	-1.06401743	1.97563111
C	4.66176496	-1.06401743	-1.97563111
C	5.00876396	2.99926570	0.00000000
C	5.46783360	-2.04599778	0.00000000
C	5.60629198	1.27959956	1.75224003
C	5.60629198	1.27959956	-1.75224003
C	5.81407301	-0.52902379	0.00000000
C	-0.77799290	5.78602093	0.00000000
C	-1.80951301	-5.55058320	0.00000000
C	-2.07468058	-4.30811863	1.97563111
C	-2.07468058	-4.30811863	-1.97563111
C	-2.49503465	5.18099284	1.75224003
C	-2.49503465	5.18099284	-1.75224003
C	-3.21140778	-4.87546626	0.00000000
C	-3.73844524	2.98131059	1.97563111
C	-3.73844524	2.98131059	-1.97563111
C	-4.03862268	4.21578439	0.00000000
C	-4.49589709	-3.58535829	1.75224003
C	-4.49589709	-3.58535829	-1.75224003
C	-4.66176496	-1.06401743	1.97563111
C	-4.66176496	-1.06401743	-1.97563111
C	-5.00876396	2.99926570	0.00000000
C	-5.46783360	-2.04599778	0.00000000

C	-5.60629198	1.27959956	1.75224003
C	-5.60629198	1.27959956	-1.75224003
C	-5.81407301	-0.52902379	0.00000000
H	0.00000000	-5.57489306	2.83003255
H	0.00000000	-5.57489306	-2.83003255
H	0.00000000	-6.83048159	1.55253120
H	1.20976726	6.79268893	-0.00000000
H	1.85727463	-6.64489960	0.00000000
H	2.41885545	5.02280509	2.83003255
H	2.41885545	5.02280509	-2.83003255
H	2.96363489	6.15405127	1.55253120
H	2.96363489	6.15405127	-1.55253120
H	4.03719991	-5.59510291	0.00000000
H	4.35862690	-3.47588897	2.83003255
H	4.35862690	-3.47588897	-2.83003255
H	4.55646051	5.18100641	0.00000000
H	5.34028555	-4.25873561	1.55253120
H	5.34028555	-4.25873561	-1.55253120
H	5.43511885	1.24053041	2.83003255
H	5.43511885	1.24053041	-2.83003255
H	6.06501561	3.28933814	0.00000000
H	6.35318350	-2.69095136	0.00000000
H	6.65922716	1.51992514	1.55253120
H	6.65922716	1.51992514	-1.55253120
H	6.89158058	-0.33207961	0.00000000
H	-0.00000000	-6.83048159	-1.55253120
H	-1.20976726	6.79268893	0.00000000
H	-1.85727463	-6.64489960	0.00000000
H	-2.41885545	5.02280509	2.83003255
H	-2.41885545	5.02280509	-2.83003255
H	-2.96363489	6.15405127	1.55253120

H	-2.96363489	6.15405127	-1.55253120
H	-4.03719991	-5.59510291	0.00000000
H	-4.35862690	-3.47588897	2.83003255
H	-4.35862690	-3.47588897	-2.83003255
H	-4.55646051	5.18100641	0.00000000
H	-5.34028555	-4.25873561	1.55253120
H	-5.34028555	-4.25873561	-1.55253120
H	-5.43511885	1.24053041	2.83003255
H	-5.43511885	1.24053041	-2.83003255
H	-6.06501561	3.28933814	0.00000000
H	-6.35318350	-2.69095136	0.00000000
H	-6.65922716	1.51992514	1.55253120
H	-6.65922716	1.51992514	-1.55253120
H	-6.89158058	-0.33207961	0.00000000
N	1.15526857	5.20774593	-1.23050847
N	-1.15526857	5.20774593	-1.23050847
N	1.15526857	5.20774593	1.23050847
N	-1.15526857	5.20774593	1.23050847
N	4.79187789	2.34375114	-1.23050847
N	3.35128155	4.15020182	-1.23050847
N	4.79187789	2.34375114	1.23050847
N	3.35128155	4.15020182	1.23050847
N	4.82010543	-2.28513606	-1.23050847
N	5.33424831	-0.03252891	-1.23050847
N	4.82010543	-2.28513606	1.23050847
N	5.33424831	-0.03252891	1.23050847
N	1.21869526	-5.19326920	-1.23050847
N	3.30041729	-4.19076471	-1.23050847
N	1.21869526	-5.19326920	1.23050847
N	3.30041729	-4.19076471	1.23050847
N	-3.30041729	-4.19076471	-1.23050847

N	-1.21869526	-5.19326920	-1.23050847
N	-3.30041729	-4.19076471	1.23050847
N	-1.21869526	-5.19326920	1.23050847
N	-5.33424831	-0.03252891	-1.23050847
N	-4.82010543	-2.28513606	-1.23050847
N	-5.33424831	-0.03252891	1.23050847
N	-4.82010543	-2.28513606	1.23050847
N	-3.35128155	4.15020182	-1.23050847
N	-4.79187789	2.34375114	-1.23050847
N	-3.35128155	4.15020182	1.23050847
N	-4.79187789	2.34375114	1.23050847
O	0.00000000	4.34390193	-3.09503177
O	0.00000000	4.34390193	3.09503177
O	3.39619929	2.70837855	-3.09503177
O	3.39619929	2.70837855	3.09503177
O	4.23499124	-0.96660911	-3.09503177
O	4.23499124	-0.96660911	3.09503177
O	1.88474841	-3.91372040	-3.09503177
O	1.88474841	-3.91372040	3.09503177
O	-1.88474841	-3.91372040	-3.09503177
O	-1.88474841	-3.91372040	3.09503177
O	-4.23499124	-0.96660911	-3.09503177
O	-4.23499124	-0.96660911	3.09503177
O	-3.39619929	2.70837855	-3.09503177
O	-3.39619929	2.70837855	3.09503177

CB7 D7h symmetry, gas-phase, 3-21G basis set

C	0.00000000	0.79304700	-5.82842200
C	0.00000000	1.81361400	5.59359400
C	0.00000000	3.24360600	4.90732700
C	0.00000000	4.06217000	-4.25359000

C	0.00000000	5.05058200	-3.01296000
C	0.00000000	5.50767100	2.07046000
C	0.00000000	5.85924800	0.52358900
C	0.00000000	-0.79304700	-5.82842200
C	0.00000000	-1.81361400	5.59359400
C	0.00000000	-3.24360600	4.90732700
C	0.00000000	-4.06217000	-4.25359000
C	0.00000000	-5.05058200	-3.01296000
C	0.00000000	-5.50767100	2.07046000
C	0.00000000	-5.85924800	0.52358900
C	1.80654300	4.39732900	3.50634700
C	1.80654300	-4.39732900	3.50634700
C	1.80690800	2.43926000	-5.06524700
C	1.80690800	-2.43926000	-5.06524700
C	1.80745900	5.47977300	-1.25043400
C	1.80745900	-5.47977300	-1.25043400
C	1.80772000	0.00000000	5.61862600
C	1.96276300	4.63249900	1.05826800
C	1.96276300	-4.63249900	1.05826800
C	1.96289200	3.71362500	-2.96182500
C	1.96289200	-3.71362500	-2.96182500
C	1.96301200	2.06252600	4.27917300
C	1.96301200	-2.06252600	4.27917300
C	1.96307500	0.00000000	-4.75122200
C	-1.80654300	4.39732900	3.50634700
C	-1.80654300	-4.39732900	3.50634700
C	-1.80690800	2.43926000	-5.06524700
C	-1.80690800	-2.43926000	-5.06524700
C	-1.80745900	5.47977300	-1.25043400
C	-1.80745900	-5.47977300	-1.25043400
C	-1.80772000	0.00000000	5.61862600

C	-1.96276300	4.63249900	1.05826800
C	-1.96276300	-4.63249900	1.05826800
C	-1.96289200	3.71362500	-2.96182500
C	-1.96289200	-3.71362500	-2.96182500
C	-1.96301200	2.06252600	4.27917300
C	-1.96301200	-2.06252600	4.27917300
C	-1.96307500	0.00000000	-4.75122200
H	0.00000000	1.22829900	-6.83578200
H	0.00000000	1.85704000	6.69011800
H	0.00000000	4.07185400	5.62722700
H	0.00000000	4.57881900	-5.22174600
H	0.00000000	6.10972200	-3.30017500
H	0.00000000	6.39344800	2.71822400
H	0.00000000	6.93800200	0.32233800
H	0.00000000	-1.22829900	-6.83578200
H	0.00000000	-1.85704000	6.69011800
H	0.00000000	-4.07185400	5.62722700
H	0.00000000	-4.57881900	-5.22174600
H	0.00000000	-6.10972200	-3.30017500
H	0.00000000	-6.39344800	2.71822400
H	0.00000000	-6.93800200	0.32233800
H	1.74105200	5.25494900	4.19078100
H	1.74105200	-5.25494900	4.19078100
H	1.74216300	2.91548200	-6.05384400
H	1.74216300	-2.91548200	-6.05384400
H	1.74429600	6.54956600	-1.49511600
H	1.74429600	-6.54956600	-1.49511600
H	1.74500300	0.00000000	6.71608400
H	2.85249200	4.14312900	3.30329200
H	2.85249200	-4.14312900	3.30329200
H	2.85264700	2.29782100	-4.77152500

H	2.85264700	-2.29782100	-4.77152500
H	2.85274900	5.16047100	-1.17739300
H	2.85274900	-5.16047100	-1.17739300
H	2.85289300	0.00000000	5.29065300
H	-1.74105200	5.25494900	4.19078100
H	-1.74105200	-5.25494900	4.19078100
H	-1.74216300	2.91548200	-6.05384400
H	-1.74216300	-2.91548200	-6.05384400
H	-1.74429600	6.54956600	-1.49511600
H	-1.74429600	-6.54956600	-1.49511600
H	-1.74500300	0.00000000	6.71608400
H	-2.85249200	4.14312900	3.30329200
H	-2.85249200	-4.14312900	3.30329200
H	-2.85264700	2.29782100	-4.77152500
H	-2.85264700	-2.29782100	-4.77152500
H	-2.85274900	5.16047100	-1.17739300
H	-2.85274900	-5.16047100	-1.17739300
H	-2.85289300	0.00000000	5.29065300
N	1.23194400	4.76961700	2.23416000
N	1.23194400	-4.76961700	2.23416000
N	1.23195600	3.23939100	4.15149600
N	1.23195600	-3.23939100	4.15149600
N	1.23195700	4.71744000	-2.33434000
N	1.23195700	-4.71744000	-2.33434000
N	1.23197600	1.11628100	-5.14511800
N	1.23197600	-1.11628100	-5.14511800
N	1.23198100	3.32636800	-4.08051400
N	1.23198100	-3.32636800	-4.08051400
N	1.23199200	5.26443200	0.05709400
N	1.23199200	-5.26443200	0.05709400
N	1.23200300	1.22655800	5.11737200

N	1.23200300	-1.22655800	5.11737200
N	-1.23194400	4.76961700	2.23416000
N	-1.23194400	-4.76961700	2.23416000
N	-1.23195600	3.23939100	4.15149600
N	-1.23195600	-3.23939100	4.15149600
N	-1.23195700	4.71744000	-2.33434000
N	-1.23195700	-4.71744000	-2.33434000
N	-1.23197600	1.11628100	-5.14511800
N	-1.23197600	-1.11628100	-5.14511800
N	-1.23198100	3.32636800	-4.08051400
N	-1.23198100	-3.32636800	-4.08051400
N	-1.23199200	5.26443200	0.05709400
N	-1.23199200	-5.26443200	0.05709400
N	-1.23200300	1.22655800	5.11737200
N	-1.23200300	-1.22655800	5.11737200
O	3.08084100	4.14801400	0.94813800
O	3.08084100	-4.14801400	0.94813800
O	3.08109300	3.32526700	-2.65238100
O	3.08109300	-3.32526700	-2.65238100
O	3.08128100	1.84769800	3.83167700
O	3.08128100	-1.84769800	3.83167700
O	3.08142600	0.00000000	-4.25500900
O	-3.08084100	4.14801400	0.94813800
O	-3.08084100	-4.14801400	0.94813800
O	-3.08109300	3.32526700	-2.65238100
O	-3.08109300	-3.32526700	-2.65238100
O	-3.08128100	1.84769800	3.83167700
O	-3.08128100	-1.84769800	3.83167700
O	-3.08142600	0.00000000	-4.25500900

S-ATE Z form, water

C	0.85912100	0.16134100	0.51384400
C	1.28604400	-0.53785900	-0.61239600
C	1.75901700	0.43042000	1.54728200
C	2.61221500	-0.95757900	-0.69210200
C	3.07243600	0.00426000	1.44913600
C	3.52342100	-0.69675100	0.32643300
C	4.96216000	-1.12555700	0.21178200
C	5.92544100	-0.12369600	-0.40070400
C	-1.36765200	0.40358700	-0.34181500
C	-2.65710000	1.06606500	0.09220900
C	-3.78175200	0.81423100	-0.90784000
C	-5.36068900	-0.71015600	0.20971200
C	-5.55948500	-2.19290100	0.49578500
C	-6.68648800	-0.05815900	-0.18697300
H	0.60786300	-0.76590400	-1.42406700
H	1.41322900	0.97157600	2.42077000
H	2.93706800	-1.50207600	-1.57319400
H	3.76279000	0.21958700	2.25858400
H	4.44356400	1.24505900	-0.77495300
H	5.04788000	-2.03007100	-0.39706100
H	5.37240600	-1.37057600	1.19522400
H	6.04665000	1.71569800	-1.25880200
H	-1.01147000	0.86038400	-1.27232100
H	-1.52477400	-0.66719100	-0.49775000
H	-1.75446900	2.65219600	0.78425300
H	-2.94483800	0.67010000	1.07387000
H	-3.39966800	0.96998900	-1.92081700
H	-4.54655100	1.58315600	-0.73557700
H	-4.63538900	-2.65258000	0.85560500
H	-4.70737300	-0.79917700	-1.70606800
H	-4.99660300	-0.22976300	1.12362100

H	-5.87239500	-2.71795600	-0.41378500
H	-6.33561300	-2.34087400	1.25188800
H	-6.56899400	1.00614000	-0.40684000
H	-7.09412400	-0.54907800	-1.07805900
H	-7.42108600	-0.15242600	0.61797400
N	5.42689700	1.03421300	-0.84676800
N	-4.30939200	-0.54310700	-0.80864400
O	7.13069400	-0.39520100	-0.47882600
O	-0.41330200	0.61292800	0.69794500
O	-2.48749000	2.48009400	0.18578900

S-ATE U form, water

C	0.71844900	1.02900000	0.05644700
C	0.95574100	-0.12393800	-0.68749500
C	1.79181100	1.80038900	0.50627800
C	2.26864900	-0.49217100	-0.97367400
C	3.08897100	1.41716700	0.21152800
C	3.35077100	0.26341800	-0.53401400
C	4.76555800	-0.16256800	-0.82191100
C	5.44942400	-1.01659300	0.23169500
C	-1.65591900	0.78219700	-0.12190200
C	-2.88945900	1.54559400	0.32034200
C	-3.67646000	-2.06330800	-0.99427400
C	-4.16945700	0.85728400	-0.13292700
C	-4.83934200	-1.51806500	-0.16207500
C	-5.39564800	-2.60553800	0.74692300
H	0.14082300	-0.73789700	-1.04818400
H	1.59283500	2.69806800	1.08070600
H	2.44587800	-1.39225500	-1.55388400
H	3.76903700	-1.12023700	1.40496700
H	3.91602900	2.02469300	0.56521400

H	4.81249800	-0.74161000	-1.74857900
H	5.16298500	-1.96958800	2.00517200
H	5.41072500	0.70851600	-0.96425100
H	-1.60992700	0.75507300	-1.21651600
H	-1.67368500	-0.24182300	0.26391200
H	-2.06685800	3.28149300	-0.01123400
H	-2.88124600	-2.42801700	-0.33420000
H	-2.88493700	1.62234200	1.41580000
H	-3.24978200	-1.30108700	-1.65176800
H	-3.67032600	-0.60422600	1.20922000
H	-4.00706100	-2.89662500	-1.62116000
H	-4.07878900	0.63138400	-1.20203400
H	-4.63982700	-2.91438600	1.47803200
H	-4.98178400	1.58344200	-0.03254900
H	-5.63460200	-1.19479500	-0.84222000
H	-5.67861800	-3.48743000	0.16580300
H	-6.27580500	-2.25480400	1.29175400
N	4.73325900	-1.39464100	1.29587100
N	-4.47708700	-0.34483000	0.64919200
O	6.63347600	-1.34562000	0.08354200
O	-0.52391500	1.47739900	0.39469300
O	-2.89572400	2.85307700	-0.24562100

R-ATE Z form, water

C	1.36765400	0.40357600	-0.34181700
C	2.65709900	1.06606300	0.09220200
C	3.78175200	0.81423100	-0.90784500
C	5.36068500	-0.71015700	0.20971700
C	5.55949600	-2.19290500	0.49576400
C	6.68648200	-0.05813700	-0.18693700

C	-0.85912000	0.16133500	0.51384300
C	-1.28604300	-0.53787000	-0.61239300
C	-1.75901700	0.43042400	1.54727800
C	-2.61221500	-0.95758700	-0.69209800
C	-3.07243600	0.00426800	1.44913300
C	-3.52342200	-0.69675000	0.32643400
C	-4.96216100	-1.12555500	0.21178400
C	-5.92544200	-0.12369300	-0.40070100
H	1.01146900	0.86036800	-1.27232500
H	1.52477900	-0.66720200	-0.49774600
H	1.75445600	2.65219300	0.78423400
H	2.94483800	0.67010500	1.07386600
H	3.39966700	0.96998400	-1.92082200
H	4.54654700	1.58316000	-0.73558500
H	4.63540100	-2.65260300	0.85556300
H	4.70738900	-0.79916900	-1.70606900
H	4.99658200	-0.22978600	1.12363200
H	5.87242600	-2.71793800	-0.41381200
H	6.33561600	-2.34088300	1.25187500
H	6.56898100	1.00616600	-0.40678200
H	7.09413400	-0.54903200	-1.07802900
H	7.42107100	-0.15241400	0.61801700
H	-0.60786100	-0.76592200	-1.42406100
H	-1.41322800	0.97158600	2.42076200
H	-2.93706800	-1.50208800	-1.57318700
H	-3.76279100	0.21960200	2.25857800
H	-4.44356300	1.24505800	-0.77495700
H	-5.04788300	-2.03006900	-0.39705700
H	-5.37240700	-1.37057200	1.19522700
H	-6.04664900	1.71569900	-1.25880300
N	4.30939800	-0.54310400	-0.80864800

N	-5.42689700	1.03421600	-0.84676600
O	0.41330500	0.61292000	0.69794400
O	2.48748100	2.48009200	0.18577400
O	-7.13069500	-0.39519600	-0.47882000

R-ATE U form, water

C	1.65592200	0.78220400	-0.12190600
C	2.88946500	1.54559400	0.32034200
C	3.67643600	-2.06331000	-0.99427400
C	4.16946100	0.85727700	-0.13292800
C	4.83932500	-1.51807700	-0.16207700
C	5.39561900	-2.60555700	0.74692100
C	-0.71844600	1.02900900	0.05644800
C	-0.95574100	-0.12392600	-0.68749800
C	-1.79180700	1.80039600	0.50628500
C	-2.26864900	-0.49215800	-0.97367400
C	-3.08896800	1.41717500	0.21153700
C	-3.35076900	0.26342900	-0.53400900
C	-4.76555800	-0.16255600	-0.82190400
C	-5.44941400	-1.01660000	0.23169200
H	1.60993200	0.75508500	-1.21652000
H	1.67368200	-0.24181700	0.26390500
H	2.06689200	3.28151300	-0.01119400
H	2.88122100	-2.42801400	-0.33419900
H	2.88494300	1.62233900	1.41580000
H	3.24976100	-1.30108200	-1.65176400
H	3.67033300	-0.60422500	1.20923100
H	4.00703000	-2.89662600	-1.62116500
H	4.07879100	0.63138000	-1.20203500
H	4.63979900	-2.91438800	1.47803800
H	4.98179100	1.58343200	-0.03255100

H	5.63458700	-1.19481900	-0.84222500
H	5.67856800	-3.48745700	0.16580200
H	6.27578800	-2.25483800	1.29174300
H	-0.14082300	-0.73788400	-1.04818900
H	-1.59282900	2.69807200	1.08071800
H	-2.44587900	-1.39224100	-1.55388600
H	-3.76902800	-1.12024000	1.40496200
H	-3.91602500	2.02469800	0.56522900
H	-4.81250200	-0.74158200	-1.74858100
H	-5.16296700	-1.96961200	2.00515900
H	-5.41072700	0.70853000	-0.96422600
N	4.47708600	-0.34483900	0.64918700
N	-4.73324300	-1.39466500	1.29585700
O	0.52391900	1.47740900	0.39468900
O	2.89573800	2.85307800	-0.24561600
O	-6.63346800	-1.34562400	0.08354200

R-ATE@CB7, A form, ONIOM, water

C	0	0.00068900	3.82534200	-3.05979900	L
C	0	0.09212500	5.39043600	-1.25436600	L
C	0	0.80264000	-4.74530300	2.59354800	L
C	0	1.29860900	-5.79133000	-0.89760400	L
C	0	1.45242300	5.18781100	0.90797800	L
C	0	2.21135900	4.89947000	-2.63327100	L
C	0	2.76624600	-3.23242900	2.62910000	L
C	0	2.81524900	-5.00633100	1.01834000	L
C	0	3.05925000	-4.02653100	-1.14324500	L
C	0	3.15741400	3.50364500	1.56828700	L
C	0	3.57551400	4.70186500	-0.46593300	L
C	0	3.90180800	3.11099300	-2.22241700	L

C	0	4.17786800	-4.19632700	0.97419200	L
C	0	4.83018500	3.78812600	-0.11801500	L
C	0	4.86353200	-0.02433400	2.37361600	L
C	0	5.15328400	-2.48903500	2.64230800	L
C	0	5.26180400	-2.88020400	-0.94079200	L
C	0	5.34714800	2.37393200	1.95826400	L
C	0	5.40159700	-0.45326900	-1.45688000	L
C	0	5.99743600	1.96490100	-1.51987400	L
C	0	6.17368300	-1.11892000	0.71133400	L
C	0	6.33270700	0.45315800	0.54174300	L
C	0	-0.69685800	-5.65410000	0.72351000	L
C	0	-0.90024600	4.37512100	0.67748400	L
C	0	-1.00008700	-5.12417100	-1.59095500	L
C	0	-1.42729400	5.03378300	-1.56051900	L
C	0	-1.43083900	-3.78538900	2.01349900	L
C	0	-2.23711800	-5.39329000	0.43425600	L
C	0	-2.44983400	3.72655400	-3.50718400	L
C	0	-3.23662400	1.37787000	-3.26917600	L
C	0	-3.31359700	4.26967800	0.00540500	L
C	0	-3.49589400	-5.05054500	-1.78433600	L
C	0	-3.80921500	-2.71816800	-2.60597400	L
C	0	-3.88309500	-3.76773800	1.55507400	L
C	0	-4.38068000	2.99900200	-1.96506900	L
C	0	-4.62766600	2.16701300	0.26614000	L
C	0	-4.81128500	-0.53781600	-3.24467900	L
C	0	-4.83886200	-1.56454800	0.93089700	L
C	0	-5.08128700	-3.31927000	-0.68756800	L
C	0	-5.23519100	1.66245600	-1.98340400	L
C	0	-5.65190000	-1.95428800	-1.26923500	L
C	0	-6.22845800	0.26829700	-0.05471100	L
H	0	0.33230700	6.46003100	-1.32506300	L

H	0	0.61503800	-4.12988100	3.48346500	L
H	0	0.96841400	-5.80046000	2.86733400	L
H	0	1.18754400	4.98588000	1.95485600	L
H	0	1.38178900	-5.75576200	-1.99209200	L
H	0	1.45494000	-6.81412800	-0.51901400	L
H	0	1.72903400	6.24560500	0.76367900	L
H	0	2.35303800	4.51171900	-3.65065100	L
H	0	2.37408900	5.98717200	-2.59273600	L
H	0	2.91677700	-6.04699100	1.35461100	L
H	0	3.78507100	5.78017700	-0.45856500	L
H	0	4.87339400	-2.21819800	3.66823500	L
H	0	4.96359800	2.37953100	2.98714300	L
H	0	5.07935900	-4.81824300	1.05016900	L
H	0	5.09261100	-2.86110200	-2.02633700	L
H	0	5.78374900	4.33032300	-0.06597400	L
H	0	5.97729900	1.76540000	-2.59925500	L
H	0	6.07194400	-3.09129700	2.62329100	L
H	0	6.16961600	-3.45473200	-0.69107800	L
H	0	6.36994300	2.77704400	1.91153100	L
H	0	6.91822700	2.49050300	-1.22747400	L
H	0	7.12343100	-1.66517600	0.78260500	L
H	0	7.34213400	0.83022800	0.75098600	L
H	0	-0.46317100	-6.67282900	1.06108700	L
H	0	-2.03018600	3.29997000	-4.42744200	L
H	0	-2.04559700	5.89393200	-1.85206800	L
H	0	-2.90135400	-6.21475900	0.73262100	L
H	0	-3.03387800	4.63579000	-3.71705600	L
H	0	-3.21394100	-5.21240500	-2.83259400	L
H	0	-3.40771200	4.18488600	1.09608000	L
H	0	-3.84527600	-3.21611700	2.50444000	L
H	0	-3.87045400	5.14433900	-0.37131000	L

H	0	-4.18337800	-0.76845100	-4.11646400	L
H	0	-4.21970900	-5.80385400	-1.44486400	L
H	0	-4.50887800	-4.67021700	1.64858700	L
H	0	-4.94889900	3.90431700	-2.21616200	L
H	0	-5.83879500	-4.09296600	-0.51001900	L
H	0	-5.86877900	-0.42729300	-3.53559500	L
H	0	-6.20022300	1.74998000	-2.49960400	L
H	0	-6.40524500	0.48597300	1.00592800	L
H	0	-6.67123900	-2.01951400	-1.67029700	L
H	0	-7.17124400	0.22748800	-0.61621300	L
N	0	0.26191400	4.90715600	0.11494200	L
N	0	0.82173600	4.63063700	-2.26733100	L
N	0	2.01687900	-4.21822400	1.97147100	L
N	0	2.34959600	-4.94308900	-0.35827900	L
N	0	2.61564000	4.35530500	0.59388300	L
N	0	3.20897800	4.24872100	-1.79927100	L
N	0	4.06183200	-3.29970300	2.11229300	L
N	0	4.10374200	-3.53822800	-0.34112000	L
N	0	4.47330700	3.23510900	1.18003800	L
N	0	4.84654000	2.82257900	-1.22721100	L
N	0	5.36833000	0.97200200	1.52571100	L
N	0	5.40329000	-1.23820100	1.93619700	L
N	0	5.44895800	-1.49149500	-0.51927500	L
N	0	5.97770500	0.66575900	-0.85671200	L
N	0	-0.07056400	-5.37352100	-0.57533300	L
N	0	-0.39118100	-4.67966700	1.76342600	L
N	0	-1.31647100	4.07676400	-2.65834000	L
N	0	-1.90003500	4.46253900	-0.29766000	L
N	0	-2.26695200	-5.19157500	-1.00752500	L
N	0	-2.51765500	-4.19119500	1.23687800	L
N	0	-3.34853700	2.73184100	-2.94664600	L

N	0	-3.90837700	3.04806100	-0.56224800	L
N	0	-4.13020900	-3.73945300	-1.70660000	L
N	0	-4.34283900	0.73624900	-2.69959900	L
N	0	-4.47677600	-2.86631800	0.57747600	L
N	0	-4.67922200	-1.66039100	-2.33117700	L
N	0	-5.42158800	1.37448000	-0.56883400	L
N	0	-5.60071900	-1.05331000	-0.12108800	L
O	0	0.36031000	3.08430800	-3.99527600	L
O	0	2.36629700	-2.48691900	3.54290200	L
O	0	2.60166600	3.13006600	2.61647700	L
O	0	2.85172600	-3.75559400	-2.33913000	L
O	0	3.76232700	2.52076300	-3.30877100	L
O	0	4.13615700	0.14608600	3.36759400	L
O	0	4.98446700	-0.53636900	-2.62967200	L
O	0	-0.74491400	-4.94787600	-2.80007300	L
O	0	-1.03006600	3.97015800	1.84990200	L
O	0	-1.40146400	-2.85043200	2.83947500	L
O	0	-2.35828200	0.86019200	-3.98375600	L
O	0	-2.96596800	-2.77169600	-3.52047200	L
O	0	-4.58432000	-1.00112600	2.01385200	L
O	0	-4.61090800	2.14040300	1.50943600	L
C	0	0.04209600	0.36166700	1.22690200	H
C	0	1.00039100	-1.26348300	-3.49472600	H
C	0	1.17097700	0.18257700	-1.36073100	H
C	0	1.32108400	-0.20724400	1.04900800	H
C	0	1.65000000	-0.05997200	-2.77354200	H
C	0	1.87008600	-0.30027100	-0.23387100	H
C	0	-0.07619000	0.79784000	-1.15727200	H
C	0	-0.65051900	0.89042100	0.11691300	H
C	0	-1.83835200	0.73094700	2.67069600	H
C	0	-2.22593700	0.55276800	4.12911600	H

C	0	-2.77474300	4.88602600	4.85386200	H
C	0	-3.13645600	3.47926400	5.35996100	H
C	0	-3.60191700	1.21990900	4.36609800	H
C	0	-4.21200700	3.54281700	6.46803300	H
H	0	1.44836600	0.82819300	-3.39090700	H
H	0	1.85083900	-0.58781900	1.92193800	H
H	0	2.73273000	-0.22765800	-2.80353000	H
H	0	2.84049900	-0.78125500	-0.35629600	H
H	0	-0.23468400	-1.65733700	-1.89258200	H
H	0	-0.42975600	-2.74973600	-3.21812700	H
H	0	-0.62825000	1.15819000	-2.02507800	H
H	0	-1.47042500	1.06247700	4.75472900	H
H	0	-1.62025300	1.36606200	0.22683200	H
H	0	-1.96188800	1.78002800	2.38715800	H
H	0	-2.03513300	4.81514700	4.04557200	H
H	0	-2.04704800	-1.41208000	3.81144600	H
H	0	-2.23111400	3.01701800	5.78498200	H
H	0	-2.38711400	5.51506700	5.66826800	H
H	0	-2.47445400	0.09192300	2.04578600	H
H	0	-3.67786700	5.37314300	4.45037500	H
H	0	-3.85170300	4.10550600	7.34287600	H
H	0	-3.95747100	0.90386700	5.36666900	H
H	0	-4.28905600	0.82683500	3.61110600	H
H	0	-4.32446700	3.03270100	3.71472600	H
H	0	-4.49145000	2.53268600	6.79990500	H
H	0	-5.11297900	4.04360000	6.07991200	H
N	0	0.01898800	-1.92587200	-2.83384300	H
N	0	-3.52072400	2.66520300	4.20815400	H
O	0	1.36055100	-1.57349800	-4.64059400	H
O	0	-0.44731700	0.34884400	2.50244400	H
O	0	-2.29677600	-0.82280200	4.54146200	H

R-ATE@CB7, B form, ONIOM, water

C	0	0.21036800	-5.62723400	0.58993300	L
C	0	0.56876100	5.60746200	-0.17395500	L
C	0	1.71449300	-5.16059700	-1.44021900	L
C	0	1.74281000	-3.43291700	-3.09908600	L
C	0	2.17224200	5.18958200	-2.13845700	L
C	0	2.30551300	-4.26575600	0.69891200	L
C	0	2.39702600	4.88961500	1.46831900	L
C	0	3.16982500	-4.52474000	-1.52093100	L
C	0	3.75346000	3.32610000	-2.62080600	L
C	0	3.79795500	2.83711000	1.29497700	L
C	0	4.00158000	4.41163700	-0.49672400	L
C	0	4.18643100	-2.88964500	-3.22120200	L
C	0	4.66240900	-3.51372000	0.30946000	L
C	0	4.69525500	-0.45490100	-3.12297000	L
C	0	4.89274900	-1.07570700	0.77844800	L
C	0	5.11840100	3.28371600	-0.65652200	L
C	0	5.62465200	-1.80865200	-1.37590800	L
C	0	5.69392800	1.27907100	0.85117700	L
C	0	5.71944100	1.78170400	-2.67763400	L
C	0	6.05654000	-0.28843100	-1.16408700	L
C	0	-0.04287300	4.47655200	1.84719400	L
C	0	-0.19955400	4.37949800	-2.06866000	L
C	0	-0.34836000	-4.77054700	-2.90984900	L
C	0	-1.00768300	5.46668800	-0.09445100	L
C	0	-1.73724700	-4.17090700	1.18479900	L
C	0	-1.86463200	-5.28346300	-0.90850800	L
C	0	-2.52120400	4.88830600	1.89844000	L
C	0	-2.53279600	-3.64136600	-2.52342600	L
C	0	-2.66846000	4.51556400	-1.77737800	L

C	0	-3.29775400	-4.71287000	-0.54807800	L
C	0	-3.89279100	2.81127600	2.13041200	L
C	0	-4.08540800	-3.43309800	1.53741400	L
C	0	-4.20345300	2.57356000	-1.64718600	L
C	0	-4.28662300	4.11893000	0.18011600	L
C	0	-4.53899900	-1.00496200	1.82379800	L
C	0	-4.97931000	-3.39497600	-1.98313200	L
C	0	-5.45379000	3.03973200	0.32759800	L
C	0	-5.48086100	-0.95475000	-1.93789500	L
C	0	-5.75947100	1.15589600	2.07850700	L
C	0	-5.78906500	-2.13302900	0.12314600	L
C	0	-6.32938100	-0.63921700	0.28754600	L
C	0	-6.33573000	1.31855500	-1.39080100	L
H	0	0.19478500	-6.67741400	0.26175000	L
H	0	0.37185300	-5.54917400	1.67292400	L
H	0	0.93634300	6.64151800	-0.11956100	L
H	0	1.67313600	-6.22826400	-1.69839600	L
H	0	2.00314800	5.00810100	-3.20835500	L
H	0	2.38622400	4.54541400	2.51116900	L
H	0	2.51209900	6.22141500	-1.95724200	L
H	0	2.76034600	5.92773300	1.39615600	L
H	0	3.85642000	-2.54015700	-4.20863700	L
H	0	3.96368800	-5.24341600	-1.76737600	L
H	0	4.40253600	5.43156300	-0.41668800	L
H	0	4.60419500	-3.51271600	1.40629500	L
H	0	4.98703100	-3.64140600	-3.31252100	L
H	0	5.44474900	-4.20028100	-0.04944200	L
H	0	5.49063000	1.07418400	1.91129600	L
H	0	5.49968600	1.87664200	-3.74917900	L
H	0	6.14841700	3.65959300	-0.58788300	L
H	0	6.46073100	-2.48431800	-1.60393500	L

H	0	6.74241000	1.57921900	0.69705900	L
H	0	6.76894400	2.02745500	-2.46097800	L
H	0	7.14328300	-0.13245300	-1.12451700	L
H	0	-0.34316500	-5.86387700	-3.06019400	L
H	0	-0.50649300	-4.24125100	-3.85912100	L
H	0	-1.55014600	6.41942300	-0.17999200	L
H	0	-1.85112700	-6.34396700	-1.19224100	L
H	0	-2.32023500	4.62401300	2.94513200	L
H	0	-2.58031700	4.08781600	-2.78526900	L
H	0	-2.93024300	5.90746200	1.81536000	L
H	0	-3.08713800	5.53725900	-1.81599300	L
H	0	-3.62607100	-3.24258100	2.51659900	L
H	0	-4.06070600	-5.47767400	-0.35321300	L
H	0	-4.63744700	5.15366400	0.06734200	L
H	0	-4.86611300	-4.20905400	1.60862500	L
H	0	-5.01419500	-3.14511600	-3.05163800	L
H	0	-5.39566500	1.03477800	3.10751500	L
H	0	-5.69803500	-4.19017400	-1.73542500	L
H	0	-6.32558900	1.35150500	-2.48808300	L
H	0	-6.45685500	3.47042100	0.43855500	L
H	0	-6.53984000	-2.90935400	0.32333600	L
H	0	-6.84104600	1.35118800	2.06282100	L
H	0	-7.31879500	1.61146900	-0.99632100	L
H	0	-7.39823300	-0.57068600	0.52798500	L
N	0	0.88989700	5.01296800	-1.46247000	L
N	0	0.95972400	-4.36548900	-2.41022500	L
N	0	1.01052900	4.84025500	0.99922500	L
N	0	1.33802000	-4.95083100	-0.04588200	L
N	0	3.03720200	-3.52677500	-2.58155800	L
N	0	3.21860900	4.25881700	-1.72671400	L
N	0	3.31918400	4.02848800	0.73716400	L

N	0	3.36349300	-3.98646500	-0.17273500	L
N	0	4.70584400	-1.71830200	-2.51894700	L
N	0	4.82451600	2.38384700	0.46005700	L
N	0	4.85774700	2.74596300	-1.99213200	L
N	0	5.01321500	-2.15878700	-0.09915600	L
N	0	5.44365300	0.03426100	0.12983600	L
N	0	5.48381400	0.38241200	-2.32706700	L
N	0	-1.10400400	-5.05462600	0.31244000	L
N	0	-1.22863000	4.85799600	1.21013000	L
N	0	-1.30959900	4.59203500	-1.23469700	L
N	0	-1.47333600	-4.42551800	-2.03836300	L
N	0	-3.01412400	-3.92299700	0.66385500	L
N	0	-3.52638300	3.95110800	1.41263600	L
N	0	-3.58576400	3.66703900	-1.02932000	L
N	0	-3.63789600	-3.91122500	-1.71222600	L
N	0	-4.70649800	-2.19024100	1.10655600	L
N	0	-5.05776700	2.31785400	1.54299800	L
N	0	-5.34836900	-2.17755400	-1.26908400	L
N	0	-5.35176800	2.28680900	-0.91429400	L
N	0	-5.52437500	-0.12031200	1.39529600	L
N	0	-6.05836400	-0.06415200	-1.03033900	L
O	0	0.05365400	3.98258200	2.98866000	L
O	0	1.36148900	-2.71597200	-4.04616300	L
O	0	2.26027900	-4.02298300	1.92063200	L
O	0	3.36098900	3.11084200	-3.78448800	L
O	0	3.43331600	2.32916900	2.37054400	L
O	0	4.14940600	-0.15469900	-4.20047600	L
O	0	4.45717700	-1.11568200	1.94277900	L
O	0	-0.19933300	3.79631400	-3.16969100	L
O	0	-1.28355500	-3.72931300	2.25981100	L
O	0	-2.49719900	-2.89410200	-3.52030300	L

O	0	-3.33327900	2.37400900	3.15562400	L
O	0	-3.71384800	-0.81673600	2.74358800	L
O	0	-3.85535300	2.03529400	-2.71554800	L
O	0	-5.21670700	-0.73838400	-3.13375000	L
C	0	0.51106100	-0.08117600	3.03637500	H
C	0	0.88824500	-1.42832000	3.16583600	H
C	0	0.99591700	0.87951000	3.94708000	H
C	0	1.35539100	0.46581500	-3.04914100	H
C	0	1.74480400	-1.80115600	4.21143900	H
C	0	1.82556700	0.47826900	4.99428100	H
C	0	2.21771900	-0.86772200	5.14590900	H
C	0	2.38319400	-1.74152800	7.59622700	H
C	0	3.09427000	-1.29558900	6.30199600	H
C	0	-0.15093400	0.51640800	-2.78575600	H
C	0	-0.75855500	-0.52344500	1.02696600	H
C	0	-0.95768000	0.72456100	-4.08207500	H
C	0	-1.53191400	0.29792500	0.00952400	H
C	0	-1.81383000	-0.50956500	-1.27013900	H
H	0	0.09968400	-0.99878800	0.53047300	H
H	0	0.51859100	-2.12491900	8.36992500	H
H	0	0.52112100	-1.59978800	6.70464500	H
H	0	0.53587500	-2.19662700	2.48820700	H
H	0	0.73887900	1.92419200	3.78849200	H
H	0	1.61281500	-0.39256000	-3.67642200	H
H	0	1.70738900	1.38529400	-3.52865600	H
H	0	1.90138800	0.35365000	-2.09953500	H
H	0	2.06410700	-2.84105900	4.25860300	H
H	0	2.19850200	1.22534000	5.69626600	H
H	0	3.73137400	-2.14368700	6.01072200	H
H	0	3.77144100	-0.48350200	6.60159600	H
H	0	-0.35067500	1.39204300	-2.15738100	H

H	0	-0.62425300	1.64977100	-4.56994500	H
H	0	-0.69088000	-1.45773300	-2.68774000	H
H	0	-0.81082600	-0.12604700	-4.76686400	H
H	0	-0.92466000	1.18218200	-0.24050200	H
H	0	-1.39165800	-1.28770500	1.49761300	H
H	0	-2.02491200	0.83635300	-3.85340300	H
H	0	-2.21508600	-1.49240100	-0.98861400	H
H	0	-2.59812500	0.02202400	-1.83420100	H
H	0	-2.75947300	0.72101500	1.49993100	H
N	0	1.03004700	-1.82457400	7.55008100	H
N	0	-0.56779500	-0.69221700	-2.02831300	H
O	0	3.03533100	-2.01435100	8.61302600	H
O	0	-0.29897700	0.40167100	2.04302500	H
O	0	-2.78957300	0.76329300	0.53481400	H

S-ATE@CB7, A form, ONIOM, water

C	0	0.69682500	-5.65409600	0.72350100	L
C	0	0.90025800	4.37509600	0.67749700	L
C	0	1.00004700	-5.12416500	-1.59096400	L
C	0	1.42732500	5.03377600	-1.56049700	L
C	0	1.43082900	-3.78540200	2.01349900	L
C	0	2.23708600	-5.39329900	0.43424100	L
C	0	2.44987300	3.72657500	-3.50717400	L
C	0	3.23662300	1.37787800	-3.26916600	L
C	0	3.31361200	4.26963700	0.00542900	L
C	0	3.49585400	-5.05055900	-1.78435500	L
C	0	3.80919300	-2.71818300	-2.60599000	L
C	0	3.88308100	-3.76776400	1.55505400	L
C	0	4.38070200	2.99898500	-1.96505200	L
C	0	4.62768600	2.16697500	0.26614900	L
C	0	4.81126000	-0.53782600	-3.24468200	L

C	0	4.83885500	-1.56457700	0.93088100	L
C	0	5.08126500	-3.31929500	-0.68759100	L
C	0	5.23519800	1.66243300	-1.98339900	L
C	0	5.65188500	-1.95431600	-1.26925500	L
C	0	6.22847000	0.26825700	-0.05471900	L
C	0	-0.00065600	3.82537200	-3.05980600	L
C	0	-0.09209200	5.39043900	-1.25434900	L
C	0	-0.80266600	-4.74528800	2.59354000	L
C	0	-1.29864300	-5.79133300	-0.89760600	L
C	0	-1.45240000	5.18781500	0.90799000	L
C	0	-2.21132500	4.89949300	-2.63326200	L
C	0	-2.76626300	-3.23240600	2.62908300	L
C	0	-2.81528100	-5.00632400	1.01834000	L
C	0	-3.05927800	-4.02653100	-1.14325000	L
C	0	-3.15740800	3.50366100	1.56829500	L
C	0	-3.57548800	4.70188000	-0.46592900	L
C	0	-3.90177400	3.11101300	-2.22242100	L
C	0	-4.17789400	-4.19631100	0.97418700	L
C	0	-4.83016100	3.78814100	-0.11802200	L
C	0	-4.86355100	-0.02430900	2.37361700	L
C	0	-5.15330100	-2.48901100	2.64230200	L
C	0	-5.26182200	-2.88018300	-0.94079500	L
C	0	-5.34715100	2.37395800	1.95825700	L
C	0	-5.40158600	-0.45324800	-1.45688000	L
C	0	-5.99740800	1.96492300	-1.51988700	L
C	0	-6.17369500	-1.11889400	0.71132700	L
C	0	-6.33271100	0.45318600	0.54173300	L
H	0	0.46313100	-6.67282400	1.06107400	L
H	0	2.03022900	3.30000600	-4.42744200	L
H	0	2.04564100	5.89392500	-1.85202000	L
H	0	2.90131500	-6.21477800	0.73259400	L

H	0	3.03392700	4.63580800	-3.71702900	L
H	0	3.21389600	-5.21241700	-2.83261200	L
H	0	3.40772000	4.18483500	1.09610400	L
H	0	3.84527400	-3.21614600	2.50442300	L
H	0	3.87046900	5.14430300	-0.37127300	L
H	0	4.18334200	-0.76845000	-4.11646100	L
H	0	4.21966200	-5.80387500	-1.44488400	L
H	0	4.50886400	-4.67024400	1.64855700	L
H	0	4.94893200	3.90429800	-2.21613000	L
H	0	5.83876800	-4.09299500	-0.51003800	L
H	0	5.86875100	-0.42729800	-3.53561000	L
H	0	6.20022000	1.74994400	-2.49961900	L
H	0	6.40526200	0.48592400	1.00592000	L
H	0	6.67122400	-2.01954700	-1.67031700	L
H	0	7.17125400	0.22743800	-0.61622300	L
H	0	-0.33226400	6.46003700	-1.32503700	L
H	0	-0.61506100	-4.12986000	3.48345100	L
H	0	-0.96844500	-5.80044200	2.86733400	L
H	0	-1.18752500	4.98588000	1.95486700	L
H	0	-1.38182400	-5.75577100	-1.99209400	L
H	0	-1.45496100	-6.81413100	-0.51901200	L
H	0	-1.72900100	6.24561100	0.76369000	L
H	0	-2.35300600	4.51175200	-3.65064600	L
H	0	-2.37405700	5.98719400	-2.59271500	L
H	0	-2.91681400	-6.04697800	1.35462400	L
H	0	-3.78504400	5.78019100	-0.45856500	L
H	0	-4.87340800	-2.21817500	3.66823000	L
H	0	-4.96360700	2.37955900	2.98713900	L
H	0	-5.07939000	-4.81822000	1.05017200	L
H	0	-5.09262900	-2.86108300	-2.02634100	L
H	0	-5.78372600	4.33033700	-0.06598800	L

H	0	-5.97726300	1.76541800	-2.59926700	L
H	0	-6.07196000	-3.09127400	2.62328700	L
H	0	-6.16963800	-3.45470500	-0.69108000	L
H	0	-6.36994100	2.77708100	1.91151400	L
H	0	-6.91819800	2.49053300	-1.22749600	L
H	0	-7.12344600	-1.66514400	0.78259600	L
H	0	-7.34214000	0.83025900	0.75096100	L
N	0	0.07052700	-5.37350700	-0.57533800	L
N	0	0.39116000	-4.67966400	1.76342100	L
N	0	1.31650500	4.07678100	-2.65833700	L
N	0	1.90005000	4.46250100	-0.29764300	L
N	0	2.26691500	-5.19157200	-1.00753900	L
N	0	2.51763700	-4.19121500	1.23687200	L
N	0	3.34856400	2.73184700	-2.94664100	L
N	0	3.90839100	3.04802700	-0.56223400	L
N	0	4.13018800	-3.73947400	-1.70662600	L
N	0	4.34282000	0.73623200	-2.69957600	L
N	0	4.47675100	-2.86634100	0.57745300	L
N	0	4.67920700	-1.66041100	-2.33119200	L
N	0	5.42161600	1.37445700	-0.56883400	L
N	0	5.60071200	-1.05334100	-0.12110400	L
N	0	-0.26189400	4.90714700	0.11495300	L
N	0	-0.82170200	4.63065800	-2.26732900	L
N	0	-2.01690000	-4.21820800	1.97145500	L
N	0	-2.34963900	-4.94310300	-0.35828500	L
N	0	-2.61562500	4.35532000	0.59389400	L
N	0	-3.20894200	4.24873500	-1.79926600	L
N	0	-4.06185000	-3.29967900	2.11228400	L
N	0	-4.10376500	-3.53821800	-0.34112500	L
N	0	-4.47329500	3.23512200	1.18003200	L
N	0	-4.84650700	2.82259400	-1.22721700	L

N	0	-5.36834500	0.97202700	1.52571200	L
N	0	-5.40330900	-1.23817500	1.93619500	L
N	0	-5.44896600	-1.49147400	-0.51927700	L
N	0	-5.97769100	0.66578400	-0.85671900	L
O	0	0.74487100	-4.94787100	-2.80008200	L
O	0	1.03006900	3.97013300	1.84991700	L
O	0	1.40147000	-2.85044900	2.83948000	L
O	0	2.35827400	0.86021600	-3.98374900	L
O	0	2.96594100	-2.77169900	-3.52048500	L
O	0	4.58432300	-1.00115900	2.01384000	L
O	0	4.61092300	2.14035100	1.50944400	L
O	0	-0.36027500	3.08435800	-3.99529900	L
O	0	-2.36630600	-2.48689400	3.54288000	L
O	0	-2.60166900	3.13008700	2.61649200	L
O	0	-2.85174600	-3.75559100	-2.33913200	L
O	0	-3.76229200	2.52078700	-3.30877700	L
O	0	-4.13617500	0.14610800	3.36759400	L
O	0	-4.98444300	-0.53635300	-2.62966700	L
C	0	0.07621500	0.79785100	-1.15725400	H
C	0	0.65053900	0.89041700	0.11693400	H
C	0	1.83835400	0.73092200	2.67072100	H
C	0	2.22593100	0.55274400	4.12914300	H
C	0	2.77479100	4.88598800	4.85384700	H
C	0	3.13648300	3.47922600	5.35996300	H
C	0	3.60192600	1.21985900	4.36611900	H
C	0	4.21201900	3.54277600	6.46805000	H
C	0	-0.04208900	0.36166600	1.22691600	H
C	0	-1.00039800	-1.26345200	-3.49472800	H
C	0	-1.17095800	0.18260300	-1.36072400	H
C	0	-1.32108300	-0.20722800	1.04901300	H
C	0	-1.64998100	-0.05993000	-2.77353800	H

C	0	-1.87007800	-0.30024200	-0.23387000	H
H	0	0.23469800	-1.65732100	-1.89260300	H
H	0	0.42973600	-2.74972100	-3.21815100	H
H	0	0.62828300	1.15820100	-2.02505400	H
H	0	1.47042900	1.06247200	4.75475300	H
H	0	1.62027700	1.36604700	0.22685900	H
H	0	1.96190000	1.78000100	2.38718100	H
H	0	2.03518400	4.81510700	4.04555400	H
H	0	2.04701500	-1.41209900	3.81147400	H
H	0	2.23113100	3.01699300	5.78497500	H
H	0	2.38716500	5.51504300	5.66824400	H
H	0	2.47445400	0.09189100	2.04581700	H
H	0	3.67792300	5.37308900	4.45036000	H
H	0	3.85170700	4.10547400	7.34288500	H
H	0	3.95747800	0.90381300	5.36669000	H
H	0	4.28905400	0.82677000	3.61112600	H
H	0	4.32451700	3.03263700	3.71475200	H
H	0	4.49144700	2.53264400	6.79993200	H
H	0	5.11300100	4.04354700	6.07994000	H
H	0	-1.44832300	0.82823300	-3.39090000	H
H	0	-1.85084700	-0.58780100	1.92193700	H
H	0	-2.73271500	-0.22758900	-2.80353100	H
H	0	-2.84049700	-0.78121300	-0.35630300	H
N	0	3.52076000	2.66515300	4.20816800	H
N	0	-0.01899100	-1.92585000	-2.83386100	H
O	0	0.44731700	0.34882900	2.50246100	H
O	0	2.29673700	-0.82282500	4.54149600	H
O	0	-1.36058000	-1.57346600	-4.64058900	H

S-ATE@CB7, B form, ONIOM, water

C	0	0.53275500	4.41965700	1.87908500	L

C	0	0.66245400	4.42836800	-2.03649400	L
C	0	1.28988400	-4.32050100	1.14435200	L
C	0	1.31682100	-5.42122000	-0.95661200	L
C	0	1.55578400	5.39795800	-0.03809400	L
C	0	2.08914400	-3.82226100	-2.56609800	L
C	0	2.79021400	-4.96086800	-0.60713000	L
C	0	3.03689500	4.66368700	1.92769400	L
C	0	3.13391300	4.34580600	-1.74028200	L
C	0	3.68756100	-3.75928100	1.47812800	L
C	0	4.19426400	2.45821300	2.12774100	L
C	0	4.40320500	-1.39810400	1.77916600	L
C	0	4.45200900	2.24803300	-1.65590700	L
C	0	4.55726000	-3.78709700	-2.06369300	L
C	0	4.71926800	3.74399600	0.19456400	L
C	0	5.31208200	-1.41204900	-1.99717800	L
C	0	5.49893700	-2.63776400	0.04965100	L
C	0	5.75920300	2.53757100	0.31631900	L
C	0	5.86392600	0.60778400	2.04212800	L
C	0	6.20512800	-1.21693200	0.22425600	L
C	0	6.42028600	0.74615300	-1.43373200	L
C	0	-0.00605100	5.65273900	-0.10362700	L
C	0	-0.18123700	-4.77207300	-2.93176100	L
C	0	-0.75955400	-5.63801200	0.55980200	L
C	0	-1.63402100	5.43346800	-2.08371200	L
C	0	-1.87441300	4.99555500	1.51602700	L
C	0	-2.19164300	-3.32395100	-3.13354300	L
C	0	-2.25666800	-5.02171400	-1.44356300	L
C	0	-2.68884100	-4.05463100	0.69834900	L
C	0	-3.31318500	3.65631600	-2.58387800	L
C	0	-3.51090900	4.74473300	-0.45867100	L
C	0	-3.52083200	3.14135100	1.32214700	L

C	0	-3.64929600	-4.25521400	-1.48732200	L
C	0	-4.58661500	-2.58189400	-3.20572300	L
C	0	-4.73233900	3.73800200	-0.65657100	L
C	0	-4.73560100	-0.09174400	-3.13442700	L
C	0	-4.93731100	-3.01759000	0.36072200	L
C	0	-4.96134400	-0.56503600	0.79491500	L
C	0	-5.39336400	2.27564400	-2.68227500	L
C	0	-5.60226500	1.84185500	0.85027800	L
C	0	-5.79110300	-1.27540600	-1.33678800	L
C	0	-6.04324200	0.29108300	-1.16456800	L
H	0	0.00525300	-4.24863400	-3.87933200	L
H	0	1.22334100	-6.47316300	-1.25642600	L
H	0	2.16572700	6.30975800	-0.11163000	L
H	0	2.82407300	4.41309600	2.97534000	L
H	0	3.01365300	3.96129100	-2.76228500	L
H	0	3.24925000	-3.52089200	2.45649300	L
H	0	3.49452800	-5.78182500	-0.41936800	L
H	0	3.53352500	5.64269100	1.84346100	L
H	0	3.64289600	5.32621600	-1.74420200	L
H	0	4.39324800	-4.60401300	1.55426300	L
H	0	4.59441300	-3.52368900	-3.12899500	L
H	0	5.18467500	4.73380500	0.09688000	L
H	0	5.20471200	-4.64876300	-1.84280900	L
H	0	5.49778800	0.51778700	3.07351700	L
H	0	6.15895500	-3.49493900	0.24039300	L
H	0	6.40000100	0.79242900	-2.53053900	L
H	0	6.80496400	2.85206800	0.42248000	L
H	0	6.96055500	0.67812700	2.01717000	L
H	0	7.27779400	-1.27243500	0.45097300	L
H	0	7.43597400	0.91680100	-1.04985200	L
H	0	-0.26774000	-5.86088700	-3.09152400	L

H	0	-0.29980600	6.70708400	-0.00478600	L
H	0	-0.82368800	-6.68226100	0.22102000	L
H	0	-0.90262400	-5.56060700	1.64531700	L
H	0	-1.47588500	5.26850500	-3.15779200	L
H	0	-1.89043500	4.60510000	2.54264500	L
H	0	-1.89137600	6.48282200	-1.87293900	L
H	0	-2.16172000	6.06041300	1.49149800	L
H	0	-2.31568400	-6.08218400	-1.72556700	L
H	0	-3.81105200	5.79284900	-0.32019700	L
H	0	-4.26748900	-2.30431300	-4.21918500	L
H	0	-4.51755400	-4.90586900	-1.66011900	L
H	0	-4.83787600	-2.99411500	1.45456100	L
H	0	-5.12079200	2.33377800	-3.74465200	L
H	0	-5.46614700	-3.24385500	-3.23093100	L
H	0	-5.46740300	1.62616600	1.91868400	L
H	0	-5.71696300	4.22515700	-0.67232100	L
H	0	-5.80594800	-3.62064900	0.05152200	L
H	0	-6.41136600	2.65588000	-2.50894600	L
H	0	-6.60650200	2.24337300	0.64662000	L
H	0	-6.70868300	-1.86187500	-1.48460100	L
H	0	-7.10452700	0.57617100	-1.17357900	L
N	0	0.58988400	-5.15669000	0.27541200	L
N	0	0.97527300	-4.51835200	-2.06900700	L
N	0	1.73991400	4.75174500	1.25307900	L
N	0	1.78462300	4.52434300	-1.19755900	L
N	0	2.57477200	-4.15394800	0.60806300	L
N	0	3.17995400	-4.18419500	-1.77225100	L
N	0	3.95137600	3.64209600	1.42984000	L
N	0	3.96789300	3.39486500	-1.01849900	L
N	0	4.42017100	-2.58172900	1.03798700	L
N	0	5.04965200	-2.62031400	-1.33963800	L

N	0	5.29445700	1.84799400	1.52572600	L
N	0	5.47752100	-0.62531000	1.34855200	L
N	0	5.56250200	1.81818400	-0.93522700	L
N	0	5.98515200	-0.59847300	-1.08340200	L
N	0	-0.37122100	5.13529600	-1.41267200	L
N	0	-0.49632800	4.86848900	1.04013800	L
N	0	-1.45082600	-4.27128800	-2.41527400	L
N	0	-1.84321000	-4.87674200	-0.05392500	L
N	0	-2.75556500	4.58279500	-1.69931500	L
N	0	-2.85576400	4.23388400	0.74869500	L
N	0	-3.48072500	-3.32064900	-2.59604500	L
N	0	-3.71922400	-3.64535900	-0.15399800	L
N	0	-4.44499200	3.12561300	-1.95913900	L
N	0	-4.61350700	2.85487700	0.49851300	L
N	0	-4.94275300	-1.33718400	-2.52975800	L
N	0	-5.14490600	-1.64316900	-0.07856200	L
N	0	-5.34858500	0.86275700	-2.31722400	L
N	0	-5.44874900	0.57226100	0.14322700	L
O	0	0.40462100	3.89916500	3.00475500	L
O	0	0.61351300	3.87920000	-3.15308900	L
O	0	0.87848800	-3.85416200	2.22532100	L
O	0	2.10144000	-3.06977300	-3.56039000	L
O	0	3.59533900	2.06913400	3.15009500	L
O	0	3.62040300	-1.13474700	2.71695700	L
O	0	4.03960400	1.76808400	-2.72966500	L
O	0	5.07125400	-1.15733800	-3.19038500	L
O	0	-1.78578000	-2.66956700	-4.11470700	L
O	0	-2.57997800	-3.80257300	1.91432300	L
O	0	-2.91523400	3.41066000	-3.74025700	L
O	0	-3.23743900	2.59155000	2.40111800	L
O	0	-4.17697500	0.11660600	-4.22701200	L

O	0	-4.52555400	-0.62323700	1.95835800	L
C	0	0.12054500	0.52246600	-2.75609300	H
C	0	0.71177800	-0.55287700	1.03642800	H
C	0	0.97137200	0.75162900	-4.02096700	H
C	0	1.54253100	0.22366600	0.02819900	H
C	0	1.76131000	-0.58597100	-1.26167700	H
C	0	-0.47913800	-0.05440000	3.08119100	H
C	0	-0.82943800	0.91320000	4.04467500	H
C	0	-1.01393200	-1.35128300	3.15796400	H
C	0	-1.37427300	0.46879500	-3.07916900	H
C	0	-1.68833000	0.56683600	5.08847700	H
C	0	-1.89745000	-1.66540800	4.20012900	H
C	0	-2.24186400	-0.72660100	5.18395400	H
C	0	-2.48305800	-1.69748600	7.59117300	H
C	0	-3.14960500	-1.10056500	6.33461800	H
H	0	0.29814600	1.38525200	-2.10144900	H
H	0	0.59303700	-1.46611800	-2.67598300	H
H	0	0.65271200	1.68144500	-4.50930100	H
H	0	0.84874700	-0.09176500	-4.71965700	H
H	0	1.00324000	1.15523700	-0.20765300	H
H	0	1.27414000	-1.39185500	1.46814700	H
H	0	2.03055200	0.86554100	-3.75973600	H
H	0	2.11291800	-1.59159700	-0.99349800	H
H	0	2.56676200	-0.09288400	-1.83099700	H
H	0	2.80831000	0.52473600	1.51694300	H
H	0	-0.19728800	-0.92368600	0.54199000	H
H	0	-0.44641100	1.92415600	3.92512900	H
H	0	-0.62634000	-1.72799200	6.67763800	H
H	0	-0.66923400	-2.33908800	8.31267500	H
H	0	-0.76044900	-2.12672800	2.44558700	H
H	0	-1.60501900	-0.40054600	-3.70275800	H

H	0	-1.70948300	1.37765900	-3.58903200	H
H	0	-1.95572700	1.31864700	5.83231900	H
H	0	-1.96017900	0.36965300	-2.15232200	H
H	0	-2.33770900	-2.66095000	4.20718700	H
H	0	-3.71593200	-0.22709900	6.68697700	H
H	0	-3.89147100	-1.84687200	6.01431600	H
N	0	0.50011200	-0.70315400	-2.00786600	H
N	0	-1.14957100	-1.93547300	7.51874600	H
O	0	0.36600800	0.37580100	2.09311700	H
O	0	2.83443600	0.58171200	0.55238000	H
O	0	-3.15268800	-1.94721100	8.60251200	H
